# A Multifunctional Delivery System for Remodulating Cell Behaviors of Circulating Malignant Cells to Prevent Cell Fusion

**DOI:** 10.1002/advs.202303309

**Published:** 2023-08-17

**Authors:** Di Han, Xiao‐Yan He, Yun Huang, Min Gao, Tao Guo, Xiao‐He Ren, Xin‐Ru Liao, Xue‐Si Chen, Xuan Pang, Si‐Xue Cheng

**Affiliations:** ^1^ Key Laboratory of Biomedical Polymers of Ministry of Education Department of Chemistry Wuhan University Wuhan Hubei 430072 China; ^2^ School of Life Sciences Anhui Medical University Hefei Anhui 230011 China; ^3^ Department of Respiratory and Critical Care Medicine The First Affiliated Hospital of Anhui Medical University Anhui Public Health Clinical Center Hefei Anhui 230011 China; ^4^ Department of Thyroid and Breast Surgery The First Affiliated Hospital of Anhui Medical University Anhui Public Health Clinical Center Hefei Anhui 230011 China; ^5^ Key Laboratory of Polymer Ecomaterials Changchun Institute of Applied Chemistry Chinese Academy of Sciences Changchun Jilin 130022 China

**Keywords:** cell fusion, circulating malignant cells, functional vectors, hybrid cells, targeting delivery

## Abstract

Cell fusion plays a critical role in cancer progression and metastasis. However, effective modulation of the cell fusion behavior and timely evaluation on the cell fusion to provide accurate information for personalized therapy are facing challenges. Here, it demonstrates that the cancer cell fusion behavior can be efficiently modulated and precisely detected through employing a multifunctional delivery vector to realize cancer targeting delivery of a genome editing plasmid and a molecular beacon‐based AND logic gate. The multifunctional delivery vector decorated by AS1411 conjugated hyaluronic acid and NLS‐GE11 peptide conjugated hyaluronic acid can specifically target circulating malignant cells (CMCs) of cancer patients to deliver the genome editing plasmid for epidermal growth factor receptor (EGFR) knockout. The cell fusion between CMCs and endothelial cells can be detected by the AND logic gate delivered by the multifunctional vector. After EGFR knockout, the edited CMCs exhibit dramatically inhibited cell fusion capability, while unedited CMCs can easily fuse with human umbilical vein endothelial cells (HUVEC) to form hybrid cells. This study provides a new therapeutic strategy for preventing cancer progression and a reliable tool for evaluating cancer cell fusion for precise personalized therapy.

## Graphical abstract

Using a multifunctional delivery vector, the genome editing plasmid can be specifically delivered into circulating malignant cells in whole blood from cancer patients for robust epidermal growth factor receptor (EGFR) knockout to effectively block cancer cell fusion. This study not only demonstrates a new therapeutic strategy to prevent cancer progression through remodulating malignant cell behaviors but also provides a reliable tool for evaluating cancer cell fusion.

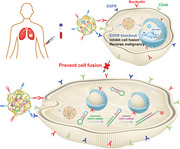

## 1 Introduction

Cell‐cell fusion is a critical cellular event in tissue development and repair as well as disease progression.^[^ ^1^ ^]^ During cancer progression, cancer cells are apt to fuse with other lineages of cells, such as endothelial cells, epithelial cells, and immune cells, to generate malignant hybrid cells. With the genomes of both parental cells, hybrid cells may exhibit new properties such as increased anti‐apoptosis ability, immune evasion, metastatic potential, treatment resistance, and cancer stem cell properties.^[^ ^2^, ^3^, ^4^ ^]^

Inhibiting cancer cell fusion and reducing the malignancy of fused cells are considered as promising strategies in cancer treatments.^[^ ^5^ ^]^ For example, antibiotic minocycline and SB‐3CT (an MMP‐9 inhibitor) can impair TNF‐α‐mediated cell fusion between breast epithelial cells and breast cancer cells.^[^ ^6^, ^7^ ^]^ Interleukin 4 receptor (IL‐4R) antibody can inhibit myoprogenitor‐rhabdomyosarcoma fusion and block tumor progression.^[^ ^8^ ^]^ XAV‐939 can reduce the malignancy of macrophage/cancer fused cells through inhibiting Wnt/β‐catenin signaling pathway.^[^ ^9^ ^]^

Although cell fusion is a critical player in cancer progression, the study on inhibition of cell fusion to interfere with cancer progression is very rare and the details are yet largely unknown. Since the main hurdle in this field is the difficulty in effective modulation of cell fusion, the purpose of this study is to construct an effective and convenient platform based on a multifunctional delivery vector to modulate of the cell fusion behavior by genome editing and investigate the cancer cell fusion behavior by using circulating malignant cells (CMCs) from cancer patients. The multifunctional delivery vector we prepared can specifically deliver a genome editing plasmid into CMCs in the blood sample of cancer patients for epidermal growth factor receptor (EGFR) knockout. The effects of EGFR knockout on inhibition of cancer cell fusion can be precisely probed at single cell resolution by using the same multifunctional delivery vector to deliver a molecular beacon‐based AND logic gate into fused cells.

As compared with chemical inhibitors, genome editing has the advantages of high specificity and persistent therapeutic benefits.^[^ ^10^ ^]^ Herein, EGFR was selected as a representative target involved in cell fusion for genome editing. As a classic target of anticancer treatments, EGFR is commonly overexpressed in different carcinomas, exerting a crucial role in the pathogenesis and progression of in diverse cancers such as non‐small cell lung cancer (NSCLC).^[^ ^11^, ^12^ ^]^ Chemical inhibitors of EGFR have been adopted as the first‐line therapy of EGFR‐mutant NSCLC. However, chemical inhibitors suffer from their critical limitations such as the mutation dependent therapeutic sensitivity and susceptibility to acquired drug resistance.^[^ ^12^, ^13^ ^]^ Genome editing can overcome the limitations of small molecule inhibitors and target both non‐mutant EGFR and mutant EGFR to exert therapeutic actions.

Our study shows that EGFR knockout not only inhibits the cancer cell proliferation, invasion and migration but also dramatically prevents malignant cell fusion. In contrast to unedited malignant cells with strong fusogenicity to fuse with endothelial cells, genome edited malignant cells can hardly fused with endothelial cells.

As far as we know, the antitumor therapeutic strategy based on genome editing to inhibit CMC fusion and the diagnostic tool for timely monitoring of the fusion between CMCs and other cell types have never been reported. Our study not only develops a new therapeutic strategy to eliminate malignant cell fusion mediated cancer progression but also provides an efficient and reliable tool for precise evaluation on the effectiveness of therapeutic actions using CMCs from cancer patients, which is of crucial importance for the personalized therapy.

## 2 Results and Discussion

### 2.1 Construction of Multifunctional Delivery Systems Loaded with Genome Editing Plasmid and Molecular Beacons

**Scheme 1:**
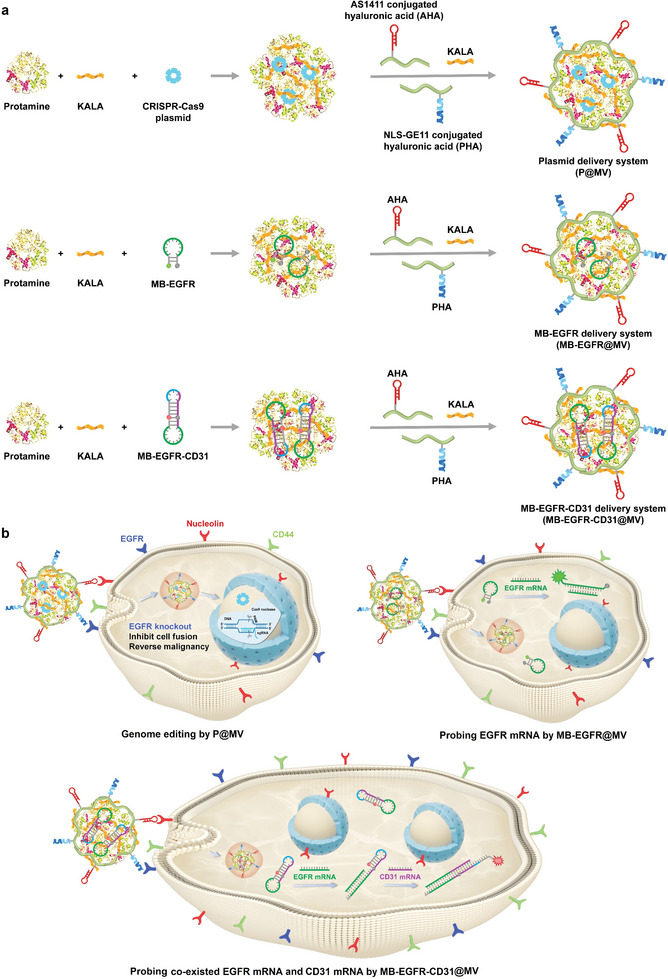
Plasmid and molecular beacon delivery systems for remodulation on malignant cell behaviors and detection on target mRNAs. a) Schematic diagram showing preparation and structures of the multifunctional plasmid delivery system (P@MV) and molecular beacon delivery systems (MB‐EGFR@MV and MB‐EGFR‐CD31@MV). b) Schematic diagram showing genome editing mediated by P@MV for EGFR knockout, probing of intracellular EGFR mRNA by MB‐EGFR@MV, and probing of co‐existed intracellular EGFR mRNA and CD31 mRNA by MB‐EGFR‐CD31@MV.

**Figure 1:**
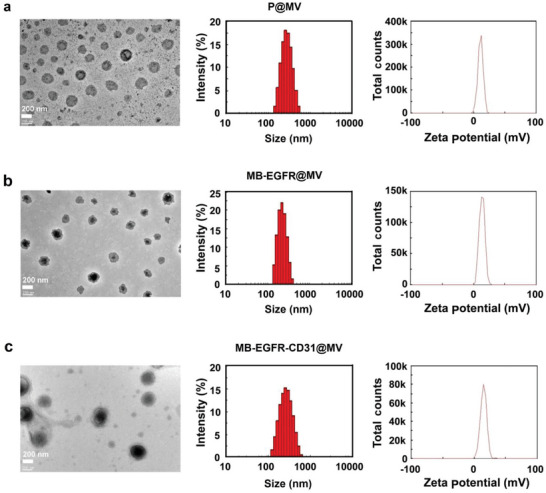
Characterizations on plasmid and molecular beacon delivery systems. TEM images, particle sizes, and zeta potentials of a) P@MV, b) MB‐EGFR@MV, and c) MB‐EGFR‐CD31@MV.

To realize efficient and cancer specific genome editing and evaluate the therapeutic efficiency, we constructed a series of nano‐systems with a cancer targeting multifunctional vector (MV) for delivery of various cargoes, including a CRISPR‐Cas9 plasmid delivery system (P@MV) for EGFR knockout, a molecular beacon delivery system (MB‐EGFR@MV) for probing EGFR mRNA, and an AND logic gate delivery system (MB‐EGFR‐CD31@MV) for probing co‐existed EGFR mRNA and CD31 mRNA to identify fused cells (**Scheme** **1**a). The TEM images, hydrodynamic sizes, and zeta potentials of these multifunctional delivery systems are shown in **Figure** **1**. All delivery systems have the sizes <300 nm with positive zeta potentials. The encapsulation efficiencies of the delivery systems are higher than 90% (Table S1, Supporting Information).

As it is well known, biosafety is the most important concern for the design of delivery vectors of nucleic acid‐based therapeutic agents.^[^ ^14^ ^]^ The delivery vectors based on biomacromolecules such as proteins, polysaccharides and DNA nanoclews possess ideal biocompatibility and biodegradability.^[^ ^15^, ^16^, ^17^ ^]^ In this study, MV consists of protamine for loading plasmid and promoting nuclear translocation,^[^ ^18^ ^]^ hyaluronic acid for targeting CD44 overexpressed on the malignant cell surface,^[^ ^19^ ^]^ AS1411 conjugated on hyaluronic acid for targeting nucleolin overexpressed cancer cell membranes and nuclei,^[^ ^20^ ^]^ NLS‐GE11 fusion peptide conjugated on hyaluronic acid (Scheme S1, Supporting Information) for targeting EGFR by the GE11 sequence^[^ ^21^, ^22^ ^]^ and enhancing nuclear transport by the NLS sequence, and a cell penetrating peptide KALA for promoting cell uptake and endosomal escape.^[^ ^23^ ^]^ Due to the co‐existence of these functional components, gene editing plasmid can be effectively delivered into cell nuclei for EGFR knockout.

By using the same multifunctional vector, molecular beacons are delivered into living cells to probe target mRNAs (Scheme 1b). The cell fusion can be detected based on co‐existed EGFR mRNA and CD31 mRNA probed by the molecular beacon‐based AND logic gate (MB‐EGFR‐CD31). MB‐EGFR‐CD31 is composed of a double‐loop‐stem structure labelled with a fluorophore and a quencher (Scheme S2, Supporting Information). In a fused cancer/endothelial cell, EGFR mRNA from the cancer cell hybridizes with its complementary sequence in MB‐EGFR‐CD31 to open the first stem‐loop structure and expose part of the stem sequence as a toehold region. CD31 mRNA from the endothelial cell further binds the toehold to open the second stem‐loop structure, resulting in separation of the fluorophore and the quencher to induce fluorescence emission.

Before living cell probing, the satisfactory hybridization performance and specificity of free molecular beacons in a buffer solution were confirmed, i.e., the free molecular beacons (MB‐EGFR for probing EGFR mRNA, and MB‐EGFR‐CD31 for probing co‐existed EGFR mRNA and CD31 mRNA) could respond to their target mRNAs specifically (Figures S1 and S2, Supporting Information). In a buffer solution, MB‐EGFR@MV and MB‐EGFR‐CD31@MV with the molecular beacons loaded in the delivery vector almost do not generate detectable fluorescence signals, indicating the encapsulation in MV can well protect the molecular beacons to avoid undesirable hybridization before cellular uptake.

### 2.2 Evaluation on the Cellular Delivery Efficiency of Different Composed Delivery Vectors

To evaluate the effects of the functional components on cellular delivery efficiency, the delivery vectors without particular components were prepared (Supplementary Materials and Methods, and Table S1, Supporting Information). Confocal laser scanning microscopy (CLSM) and flow cytometry demonstrate that the plasmid delivery system with the multifunctional vector, P@MV, exhibits the highest cellular delivery efficiency in A549 cancer cells (Figure S3, Supporting Information). The monitoring on the cellular delivery process of P@MV further indicates P@MV can effectively mediate the cellular delivery of the plasmid (Figure S4, Supporting Information). In addition, P@MV leads to the most efficient delivery in EA.hy926 hybrid cells which are derived from the fusion of A549 with HUVEC (Figure S5, Supporting Information). In normal cells such as HUVEC, P@MV also possesses the highest delivery efficiency (Figure S6, Supporting Information). However, the intracellular plasmid concentration in HUVEC is much lower than that in malignant cells. Since nucleolin and EGFR are overexpressed in A549 and EA.hy926 cells (Figure S7, Supporting Information), the presence of AS1411 and/or NLS‐GE11 in the multifunctional vector results in the enhanced cellular delivery capability because of the specific affinity of AS1411 to nucleolin as well as the interaction between GE11 and EGFR in A549 cells and EA.hy926 cells. HUVEC also expresses nucleolin; nevertheless, the expression level is relatively low, leading to limited enhancement in the delivery efficiency. The decoration of the cell penetration peptide, KALA, onto the out layer of nanoparticles leads to further increased intracellular plasmid accumulation.

In addition, we compared the genome editing mediated by different delivery systems. In consistence with the cellular uptake study, P@MV possessing the optimized composition results in the highest Cas9 expression (Figure S8, Supporting Information), the most efficient EGFR knockout (Figure S9, Supporting Information), and the most effective cell growth inhibition (Figure S10, Supporting Information) in cancer cells (A549) and cancer/endothelial hybrid cells (EA.hy926). The genome editing efficiency of the system with NLS‐GE11 decoration is higher than its counterpart without the fusion peptide decoration because of the enhanced cellular uptake and nuclear transportation.

### 2.3 Remodulation on Cellular Behaviors of Malignant Cell Lines to Reduce Malignancy and Prevent Cell Fusion

By using the most efficient multifunctional plasmid delivery system, P@MV, we exerted genome editing in malignant cell lines, including a cancerous cell line (A549) and a cancer/endothelial hybrid cell line (EA.hy926), to study the effects of EGFR knockout on cell behaviors. Both A549 and EA.hy926 cell lines are EGFR overexpressed. As an A549/HUVEC hybrid cell line, EA.hy926 has slightly lower EGFR expression as compared with A549, and lower CD31 expression as compared with HUVEC (Figure S11, Supporting Information).

**Figure 2:**
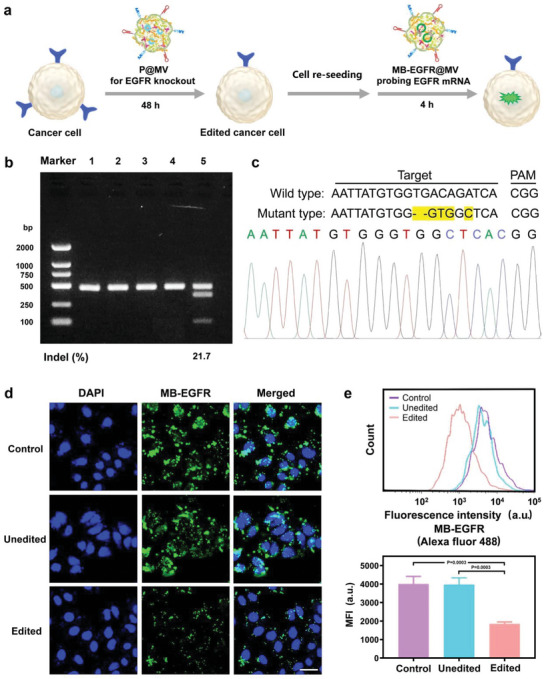
Genome editing by the plasmid delivery system and probing of EGFR mRNA by the molecular beacon delivery system in a cancerous cell line. a) Schematic diagram showing the procedure of genome editing and EGFR mRNA detection in cancerous A549 cells. b) T7E1 assay on the DNA mutation in A549 cells after different treatments: 1) untreated control, 2) MV treatment without rehybridization with the wild type, 3) P@MV treatment without rehybridization with the wild type, 4) MV treatment with rehybridization with the wild type, and 5) P@MV treatment with rehybridization with the wild type. c) The DNA sequencing result of edited A549 cells. d) CLSM observation on EGFR mRNA probed by MB‐EGFR@MV in A549 cells. Cell nuclei were stained by DAPI. Scale bar: 30 µm. e) Flow cytometry analysis on A549 cells with intracellular EGFR mRNA probed by MB‐EGFR@MV. Data are given as mean ± s.d., n = 3. The results were statistically analyzed using one‐way ANOVA.

First, the genome editing was carried out in A549 cells (**Figure** **2**a). After being co‐incubation with P@MV, the T7 endonuclease I (T7E1) assay (Figure 2b) and DNA sequencing (Figure 2c) demonstrate the successful genome editing mediated by P@MV.

The molecular beacon delivery system, MB‐EGFR@MV, was used to probe the EGFR mRNA change in living cells after genome editing. As observed by CLSM (Figure 2d) and quantitated by flow cytometry (Figure 2e), EGFR overexpressed unedited cancerous A549 cells exhibit strong fluorescence signals generated via hybridization between MB‐EGFR and intracellular EGFR mRNA. While edited A549 cells have a dramatically decreased fluorescence intensity due to the EGFR knockout by P@MV. qPCR confirms that genome editing significantly downregulates the mRNA level of EGFR (Figure S12, Supporting Information). These results verify that our plasmid delivery system can realize efficient EGFR knockout and the molecular beacon delivery system can accurately probe EGFR mRNA in living cells.

**Figure 3:**
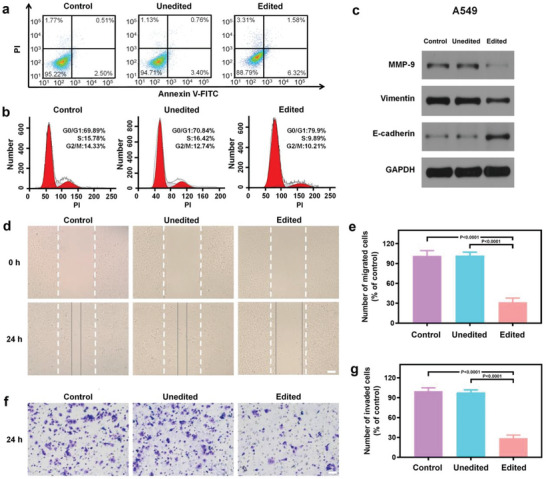
Inhibition of cell growth, migration, and invasion by EGFR knockout in a cancerous cell line. a) The cell apoptosis of unedited and edited A549 cells assessed by Annexin V/PI protocol. b) The cell cycles of unedited and edited A549 cells. c) Western blotting analysis on metastasis related proteins in unedited and edited A549 cells. d) Wound healing assay on unedited and edited A549 cells. Scratched lines are marked by white lines, and the boundary position of most migrated A549 cells is marked by the grey lines. Scale bar: 50 µm. e) The number of migrated unedited and edited A549 cells. Data are given as mean ± s.d., n = 3. The results were statistically analyzed using one‐way ANOVA. f) Transwell invasion assay on unedited and edited A549 cells. Scale bar: 50 µm. g) The number of invaded unedited and edited A549 cells. Data are given as mean ± s.d., n = 3. The results were statistically analyzed using one‐way ANOVA. The unedited cells were treated by the blank vector MV and the edited cells were treated by P@MV for 48 h. Untreated cells were served as a control.

Annexin V‐FITC/PI apoptosis analysis depicts that the number of early and late apoptotic cells after genome editing is obviously increased compared with unedited cells treated by the blank vector MV and the untreated cells (**Figure** **3**a). The cell cycle assay demonstrates that genome editing leads to the cell cycle arrest at G0/G1 phase with reduced cell populations at G2/M and S phases (Figure 3b). The above results are in consistent with previous studies indicating that inhibition of EGFR results in apoptosis as well as cell cycle arrest.^[^ ^24^ ^]^

Metastasis and invasion are the basic features of malignant tumors, leading to poor prognosis. Therefore, we investigated the effects of EGFR knockout on the proteins promoting cell migration and invasion. Western blotting indicates that the edited cells possess decreased matrix metalloproteinase‐9 (MMP‐9) and vimentin, and elevated E‐cadherin (Figure 3c). During cancer progression, MMP‐9 degrades the extracellular matrix to promote invasion and metastasis.^[^ ^25^ ^]^ Vimentin commonly overexpressed in various epithelial cancers plays a pivotal role during EMT, correlating with enhanced invasion and poor prognosis.^[^ ^26^ ^]^ E‐cadherin contributes to the formation of adhesive intercellular junctions between epithelial cells to restrict the cell migration.^[^ ^27^ ^]^ The reduced MMP‐9 and vimentin and enhanced E‐cadherin in edited cancer cells suggest that downregulation of EGFR can prevent cancer metastasis by suppressing EMT, as well as inhibiting cancer cells migration and invasion. The wound healing assay (Figure 3d,e) and transwell assay (Figure 3f,g) confirm that the migration and invasion of A549 cells are significantly suppressed after genome editing. These results are in good consistence with previous studies indicating that EGFR inhibition results in inhibited cell migration and invasion.^[^ ^28^ ^]^

As it is well known, circulating tumor cells (CTCs) and other CMCs such as circulating hybrid cells (CHCs) co‐exist in the blood of cancer patients.^[^ ^29^, ^30^, ^31^, ^32^ ^]^ In this study, the genome editing was also exerted in a cancer/endothelial hybrid cell line (EA.hy926).^[^ ^33^ ^]^ As expected, the genome editing leads to efficient EGFR knockout in hybrid EA.hy926 cells (Figures S13 and S14, Supporting Information). After EGFR knockout, edited EA.hy926 cells exhibit significantly suppressed cell growth, migration, and invasion (Figure S15, Supporting Information).

The fusogenicity of cancer cells is closely related to cancer progression, drug resistance and cancer heterogeneity.^[^ ^34^, ^35^, ^36^ ^]^ In this investigation, we confirmed that untreated A549 cells could fused with HUVEC to form hybrid cells by staining two types of cells before co‐incubation (Figure S16, Supporting Information).

**Figure 4:**
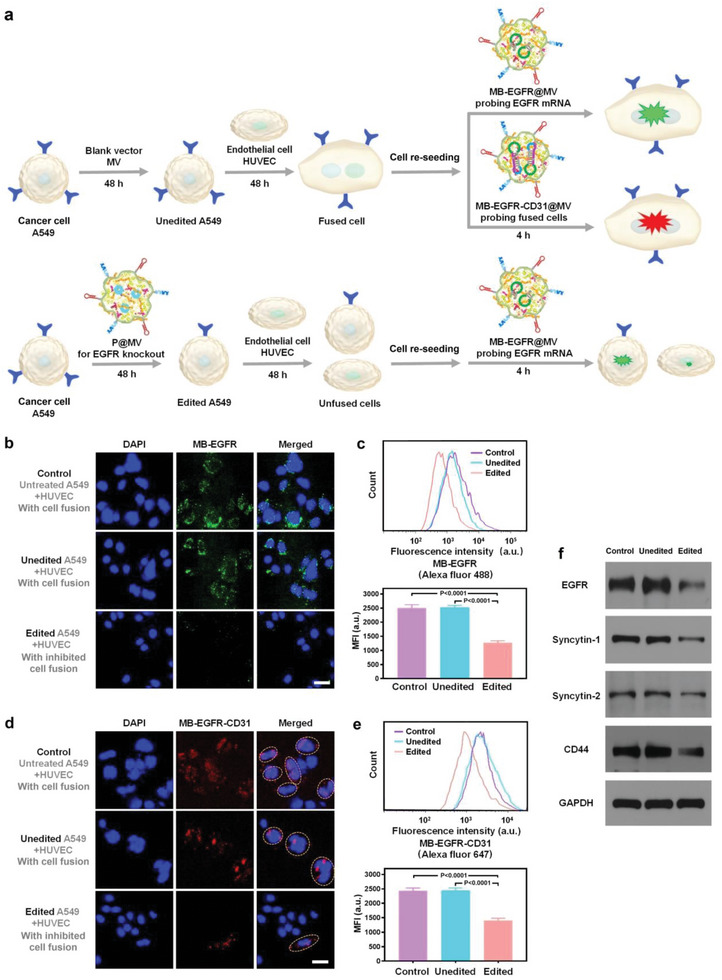
Inhibition of cell fusion by EGFR knockout in a cancerous cell line. a) Schematic diagram showing evaluation on cell fusion of unedited and edited cancer cells (A549) with HUVEC. b) CLSM observation on EGFR mRNA probed by MB‐EGFR@MV in mixed cells. Cell nuclei were stained by DAPI. Scale bar: 30 µm. c) Flow cytometry analysis on mixed cells with intracellular EGFR mRNA probed by MB‐EGFR@MV. Data are given as mean ± s.d., n = 3. The results were statistically analyzed using one‐way ANOVA. d) CLSM observation on co‐existed EGFR mRNA and CD31 mRNA probed by MB‐EGFR‐CD31@MV in mixed cells. Cell nuclei were stained by DAPI. Scale bar: 30 µm. e) Flow cytometry analysis on mixed cells with co‐existed intracellular EGFR mRNA and CD31 mRNA probed by MB‐EGFR‐CD31@MV. Data are given as mean ± s.d., n = 3. The results were statistically analyzed using one‐way ANOVA. f) Western blotting analysis on fusion related proteins in unedited and edited A549 cells. The unedited cells were treated by the blank vector MV and the edited cells were treated by P@MV for 48 h. Untreated cells were served as a control.

To study the effect of genome editing on cancer cell fusogenicity, edited and unedited cancerous A549 cells, respectively, were co‐incubated with endothelial cells for 48 h. After cell re‐seeding, the molecular beacon delivery systems were added to mixed cells, followed by co‐incubation for 4 h to allow the cellular delivery of molecular beacons and hybridization with target mRNAs (**Figure** **4**a). Being consistence with the result of EGFR knockout in A549 cells, the probing of MB‐EGFR@MV shows markedly reduced fluorescence signals in edited A549 mixed with HUVEC as analyzed by CLSM and flow cytometry (Figure 4b,c), indicating that the downregulated EGFR mRNA level in the edited A549 cells is not affected by co‐incubation with HUVEC.

Herein, MB‐EGFR‐CD31@MV was used to evaluate the cell fusion. MB‐EGFR‐CD31@MV does not induce detectable fluorescence emission in A549 cells or HUVEC, while emits strong fluorescence signals in hybrid EA.hy926 cells (Figure S17, Supporting Information). As compared with the strong red fluorescence emitted by MB‐EGFR‐CD31 hybridized with co‐existed EGFR mRNA and CD31 mRNA in the fused cells after co‐incubating unedited A549 cells with HUVEC, dramatically reduced red fluorescence is observed in co‐incubated edited A549 cells and HUVEC (Figure 4d,e). This result demonstrates that unedited A549 cells can easily fuse with HUVEC and EGFR knockout efficiently prevents the fusion between edited A549 cells and HUVEC. As detected by MB‐EGFR‐CD31@MV, >70% unedited A549 cells fuse with HUVEC. While the percentage of edited A549 fused with HUVEC (<20%) dramatically decreases.

To explore the correlation between EGFR knockout and cell fusion, we examined the expression of the proteins involved in cell fusion (syncytin‐1 and syncytin‐2)^[^ ^5^, ^37^, ^38^ ^]^ and cell adhesion (CD44)^[^ ^39^, ^40^ ^]^ in unedited and edited A549 cells. Western blotting assay demonstrates that syncytin‐1, syncytin‐2 and CD44 in edited cells are significantly downregulated (Figure 4f). Clearly, EGFR knockout results in significantly reduced expression of the proteins promoting cell fusion and cell adhesion, and thus impairs the fusogenic activity of malignant cells. A previous report shows that EGFR inhibition blocks CD44 mediated cancer stem cell aggregation.^[^ ^41^ ^]^ Our results are in consistence with the previous literature, and the reduced CD44 in edited cells is favorable for preventing malignant cell aggregation.

Previous studies demonstrate that cancer growth and metastasis can be effectively inhibited through inhibiting EGFR mediated oncogenesis.^[^ ^42^ ^]^ In this study, EGFR knockout leads to the suppressed cell growth, as well as the strongly inhibited migration, invasion, and cell fusion, demonstrating the genome editing can efficiently remodulate the cell behaviors of malignant cells to prevent cancer development.

### 2.4 Remodulation on Cellular Behaviors of CMCs of Cancer Patients to Reduce Malignancy and Prevent Cell Fusion

During cancer progression, CMCs including CTCs and other malignant cells such as CHCs exert a critical role in promoting cancer development. After remodulating the behaviors of the cancer cell line and the hybrid cell line by EGFR knockout, we further carried out genome editing on CMCs (CTC and CHC as typical examples shown in the schematic diagram) from NSCLC patients (see Table S2, Supporting Information, for clinical information). Herein, the genome editing in CMCs was initiated in whole blood to mimic the in vivo environment. Based on the ex vivo experiment, P@MV can effectively deliver the loaded plasmid into CMCs in whole blood (Figure S18, Supporting Information), implying the stability of P@MV in whole blood can satisfy the genome editing requirement.

**Figure 5:**
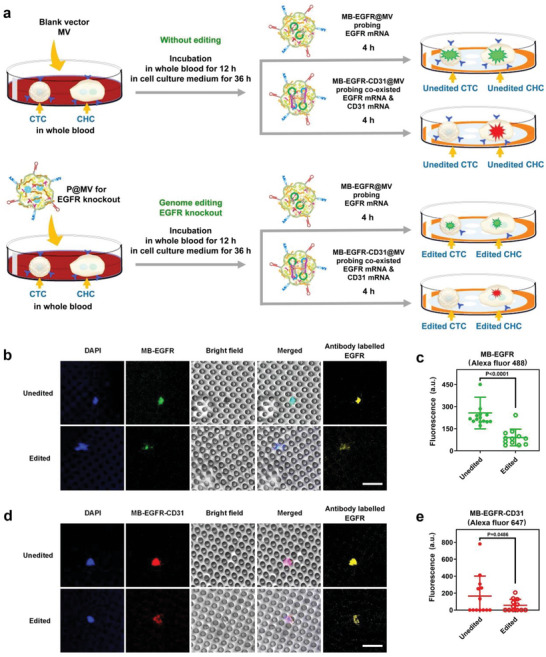
Genome editing in CMCs from a patient by the plasmid delivery system and probing of mRNAs by molecular beacon delivery systems. a) Schematic diagram showing the procedure of genome editing and mRNA detection in CMCs from the patient P1. b) CLSM observation on EGFR mRNA probed by MB‐EGFR@MV in CMCs. Cell nuclei were stained by DAPI. EGFR of CMCs was antibody labelled. Scale bar: 30 µm. c) Fluorescence of MB‐EGFR@MV probed cells analyzed by ImageJ v1.8.0 software. Data are given as mean ± s.d., n = 14 for unedited cells, and n = 12 for edited cells. All cells are shown in Figure S21 (Supporting Information). The results were statistically analyzed using Student's t‐test. d) CLSM observation on co‐existed EGFR mRNA and CD31 mRNA probed by MB‐EGFR‐31@MV in CMCs. Cell nuclei were stained by DAPI. EGFR of CMCs was antibody labelled. Scale bar: 30 µm. e) Fluorescence of MB‐EGFR‐CD31@MV probed cells analyzed by ImageJ v1.8.0 software. Data are given as mean ± s.d., n = 13 for unedited cells, and n = 12 for edited cells. All cells are shown in Figure S21 (Supporting Information). The results were statistically analyzed using Student's t‐test. The unedited CMCs were treated by the blank vector MV and the edited CMCs were treated by P@MV.

To exert genome editing and detect related mRNAs in malignant cells from patients, the multifunctional genome editing plasmid delivery system (P@MV) was added to 1 ml of unprocessed peripheral blood. After 12 h, CMCs were isolated from whole blood followed by incubation in the cell culture medium for 36 h. After that, the molecular beacon delivery systems (MB‐EGFR@MV for probing EGFR mRNA, and MB‐EGFR‐CD31@MV for probing co‐existed EGFR mRNA and CD31 mRNA, respectively) were added into the cells followed by incubation for 4 h (**Figure** **5**a). In our study, CMCs were isolated by size sieving using a filtration membrane. In addition, the MB probing further ensures the accurate identification of the isolated CMCs. The MB delivery system (MB‐EGFR@MV) can specifically deliver MB‐EGFR into CMCs to induce fluorescence emission and no false fluorescence signal is generated in blood cells in case a few blood cells are remained on the filter membrane (Figure S19, Supporting Information). The study on the CMC viability by calcein AM staining shows that CMCs are living cells after genome editing (Figure S20, Supporting Information).

CLSM observation shows much weaker green fluorescence signals induced by the hybridization of MB‐EGFR and EGFR mRNA in edited CMCs as compared with unedited CMCs (Figure 5b,c; Figure S21, Supporting Information), indicating that the level of EGFR mRNA dramatically reduces after genome editing. The antibody labelling result is consistent with the molecular beacon sensing, i.e., EGFR protein on the edited cells surface is downregulated considerably after EGFR knockout by P@MV.

In our investigation, CMCs from cancer patient are found to be heterogenic based on their expression of CD31. Both EGFR+/CD31‐ and EGFR+/CD31+ CMCs exist in the blood. As it is well known, CD31 is a biomarker of endothelial cells.^[^ ^32^, ^43^ ^]^ CD31+ CMCs may be hybrid cells generated via the heterotypic fusion of cancer cells with endothelial cells. Nevertheless, transdifferentiation of cancer cells may also form CD31+ CMCs.^[^ ^32^ ^]^ In other words, the EGFR+/CD31+ cells from patients may be fused hybrid cells or differentiated CTCs. After genome editing, the red fluorescence emitted by MB‐EGFR‐CD31 in edited EGFR+/CD31+ cells is also dramatically weaker than that in the unedited ones, which is caused by downregulated EGFR mRNA (Figure 5d,e).

**Figure 6:**
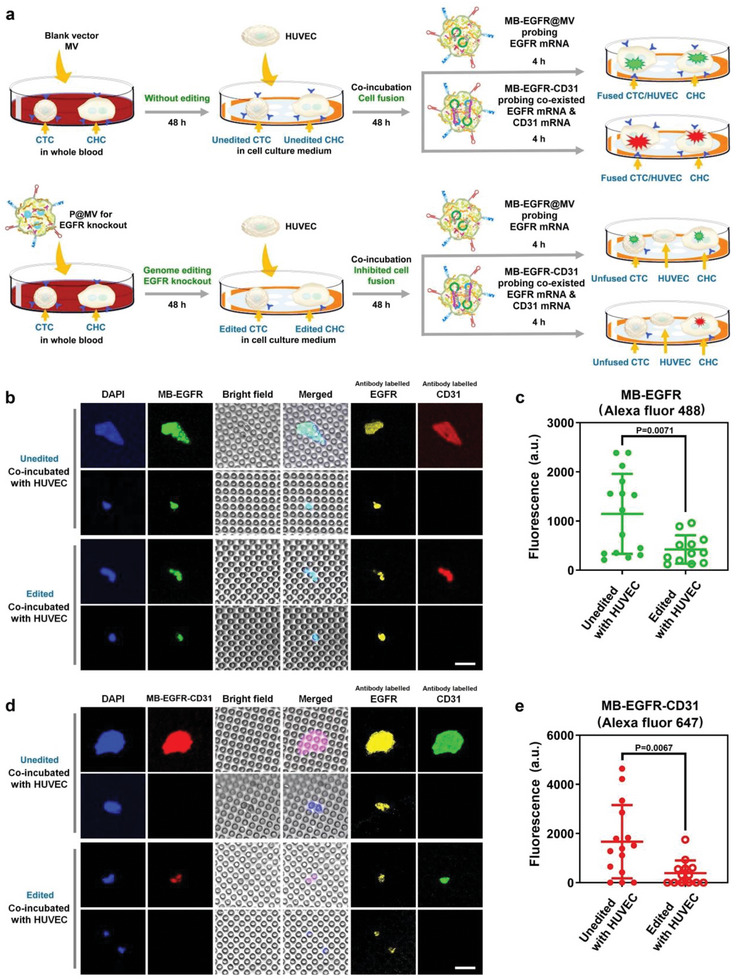
Inhibition of cell fusion by EGFR knockout in CMCs from a patient. a) Schematic diagram showing evaluation on cell fusion between unedited and edited CMCs from the patient P2 with HUVEC. b) CLSM observation on EGFR mRNA probed by MB‐EGFR@MV in mixed cells. Cell nuclei were stained by DAPI. EGFR and CD31 of the cells were antibody labelled. Scale bar: 30 µm. c) Fluorescence of MB‐EGFR@MV probed cells analyzed by ImageJ v1.8.0 software. Data are given as mean ± s.d., n = 15 for unedited cells, and n = 12 for edited cells. All cells are shown in Figure S22 (Supporting Information). The results were statistically analyzed using Student's t‐test. d) CLSM observation on co‐existed EGFR mRNA and CD31 mRNA probed by MB‐EGFR‐CD31@MV in mixed cells. Cell nuclei were stained by DAPI. EGFR and CD31 of the cells were antibody labelled. Scale bar: 30 µm. e) Fluorescence of MB‐EGFR‐CD31@MV probed cells analyzed by ImageJ v1.8.0 software. Data are given as mean ± s.d., n = 15 for unedited cells, and n = 13 for edited cells. All cells are shown in Figure S22 (Supporting Information). The results were statistically analyzed using Student's t‐test. The unedited CMCs were treated by the blank vector MV and the edited CMCs were treated by P@MV.

We further investigated the efficiency of EGFR knockout on prevention of fusion of CMCs with other types of cells, using the endothelial cell line, HUVEC, as a representative. Endothelial cells were added to unedited and edited CMCs (CTC and CHC as typical examples shown in the schematic diagram), respectively, followed by co‐incubation for 48 h. (**Figure** **6**a). As mentioned above, the EGFR+/CD31+ cells from patients may be fused hybrid cells or differentiated CTCs. In our schematic diagram showing the experimental procedure of genome editing on CMCs and evaluation on the fusogenic activity of CMCs, the EGFR+/CD31+ cell is represented by CHC. For convenience, we do not draw all possible types of EGFR+/CD31+ cells in the schematic diagram.

After co‐incubation of CMCs with HUVEC, MB‐EGFR@MV and MB‐EGFR‐CD31@MV were added to the mixed cells, respectively, for detection of intracellular mRNAs. The cells were also antibody labelled to identify EGFR and CD31 proteins in the cell surface. Typical EGFR+/CD31+ and EGFR+/CD31‐ cells in each sample observed by CLSM are shown in Figure 6b,d.

For unedited cells, after co‐incubating with HUVEC, large green fluorescent faculae induced by intracellular EGFR mRNA can be clearly visualized as detected by MB‐EGFR@MV (Figure 6b), indicating the occurrence of cell fusion between unedited CMCs with HUVEC. The large size of the fused cells implies that each malignant cell may fuse with several endothelial cells. The intensity of the green fluorescence induced by EGFR mRNA in unedited cells is higher as compared with edited cells (Figure 6c). In our study, most unedited CMCs fuse with HUVEC. Nevertheless, a few EGFR+/CD31‐ unedited CMCs without cell fusion can be observed (Figure S22, Supporting Information). For edited cells, co‐incubated with HUVEC does not lead to obvious cell fusion as indicated by much smaller fluorescent faculae with weaker green fluorescence signals. The antibody labelling is in good consistence with MB‐EGFR@MV probing, confirming the cell fusion between edited CMCs and HUVEC is dramatically inhibited by genome editing.

In addition, the detection of co‐existed EGFR mRNA and CD31 mRNA by the AND logic gate delivery system (MB‐EGFR‐CD31@MV) shows the same trend, i.e., CMC/HUVEC fusion leads to large red fluorescent faculae in unedited CMCs co‐incubated with HUVEC, while edited CMCs can hardly fuse with HUVEC (Figure 6d). Besides, after genome editing, the intensity of red fluorescence induced by co‐existed EGFR mRNA and CD31 mRNA is obviously decreased in edited CMCs co‐incubated with HUVEC (Figure 6e). Clearly, as compared with unedited CMCs with a high fusogenic activity to form giant fused cells with HUVEC, edited CMCs exhibits greatly inhibited fusogenic capability. These results demonstrate that EGFR knockout effectively prevents cell fusion between CMCs and HUVEC.

**Figure 7:**
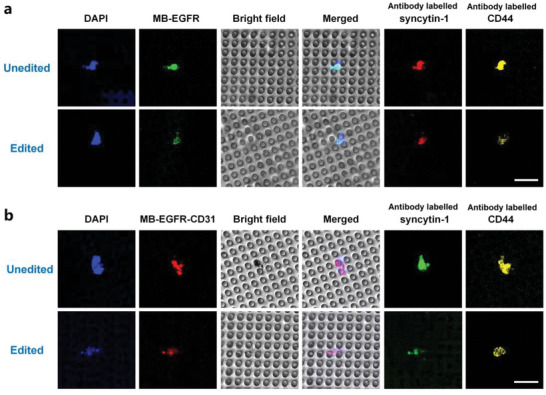
Effects of gene editing on expression of cell fusion related proteins in CMCs from a patient. a) CLSM observation on CMCs with EGFR mRNA probed by MB‐EGFR@MV. b) CLSM observation on CMCs with co‐existed EGFR mRNA and CD31 mRNA probed by MB‐EGFR‐31@MV. CMCs were from the patient P3. Cell nuclei were stained by DAPI. Syncytin‐1 and CD44 of CMCs were antibody labelled. Scale bar: 30 µm. The unedited CMCs were treated by the blank vector MV and the edited CMCs were treated by P@MV.

To further study the prevention of cell fusion by EGFR knockout, the expression of fusion related proteins in CMCs was evaluated by immunofluorescence staining. CLSM imaging shows that syncytin‐1 and CD44 in CMCs are significantly downregulated after EGFR knockout (**Figure** **7**). Obviously, the genome editing on CMCs dramatically reduces the fusogenic activity of CMCs via downregulating fusion promoting proteins. This result is in consistent with the study on the cancer cell line (A549) and the cancer/endothelial hybrid cell line (EA.hy926).

## 3 Conclusion

By using a multiple functional vector to specifically deliver the genome editing plasmid and the molecular beacon‐based AND logic gate into CMCs of cancer patients, the cancer cell fusion can be efficiently blocked through effective EGFR knockout mediated by the plasmid delivery system, and the therapeutic outcome can be directly imaged by the molecular beacon delivery system. Due to the convenience of the ex vivo study on CMCs by using a few milliliters of blood, our approach enables frequent evaluation on particular treatments to provide valuable guides for adjusting and optimizing anticancer therapy dynamically. This study provides a new and effective platform for personal precision therapy through timely and accurate evaluation on therapeutic outcomes of the anticancer treatment on particular cancer patients.

## 4 Experimental Section

### Materials

Protamine sulfate (PS) was obtained from Shanghai Yuanye Bio‐Technology Co. Ltd. (Shanghai, China). Hyaluronic acid (HA) (sodium salt, Mw = 50 kDa) was supplied by Shandong Freada Biotechnology Co. Ltd. (Shandong, China). KALA peptide (WEAKLAKALAKALAKHLAKALAKALKACEA) and Cys‐NLS‐GE11 peptide (Cys‐PKKKRKV‐YHWYGYTPQNVI) were from GL Biochem Co. Ltd. (Shanghai, China). Aminated AS1411 (5′‐NH_2_‐(CH_2_)_6_‐GGTGGTGGTGGTTGTGGTGGTGGTGG‐3′) was from Sangon Biotech Co. Ltd. (Shanghai, China).

*CRISPR‐Cas9 plasmids for EGFR knockout (EGFR Genbank accession*: NM_0 013 46898.2) were from Genomeditech (Shanghai, China). Three sgRNA sequences (5′‐ TCTGTCACCACATAATTACC‐3′, 5′‐AATTATGTGGTGACAGATCA‐3′, and 5′‐CGGCGGGCTTCCTACCTTTG‐3′) for EGFR knockout were designed by an online guide tool (http://crispr.mit.edu/). The sgRNA sequence of 5′‐AATTATGTGGTGACAGATCA‐3′ was found to be the most efficient one, and the plasmid containing this sgRNA was abbreviated as “P”.

The molecular beacon for probing EGFR mRNA coded as “MB‐EGFR” (5′‐Alexa fluor 488‐CACGCGGACGGGATCTTAGGCCCATTCGTTGGCGTG‐DABCYL‐3′), EGFR mRNA (5′‐CAACGAAUGGGCCUAAGAUCCCGUCC‐3′) and CD31 mRNA (5′‐CCUGCGGUAUUCAAAGACAACCCC‐3′) (CD31 Genbank accession: NM_000442.5) were purchased from ShineGene Molecular Biotechnology Co. Ltd. (Shanghai, China). The molecular beacon‐based AND logic gate for probing co‐existed EGFR mRNA and CD31 mRNA coded as “MB‐EGFR‐CD31” (5′‐CAACCCCGGACGGGATCTTAGGCCCATTCGTTGGGGGTTG/iBHQ2dT/CT

TTGAATACCGCAGGCGCACTGCCGGTATTCAAAGA‐Alexa fluor 647‐3′) was supplied by Sangon Biotech Co. Ltd. (Shanghai, China).

### Preparation of the Multifunctional Plasmid Delivery System

PS (30 µg), KALA (3 µg) and CRISPR‐Cas9 plasmid (2 µg) were mixed for 15 min in ultrapure water (50 µl) to form plasmid@PS/KALA nanoparticles. AHA (5 µg), PHA (5 µg) and KALA (2 µg) were mixed in ultrapure water (50 µl) to form AHA/PHA/KALA complexes, and then added dropwise to plasmid@PS/KALA nanoparticles in ultrapure water followed by mixing for 10 min to form plasmid@PS/KALA/AHA/PHA/KALA (coded as “P@MV”) nanoparticles in 100 µl of ultrapure water.

### Preparation of Molecular Beacon Delivery Systems

PS (30 µg), KALA (3 µg) and MB‐EGFR (0.07 nmol) were mixed for 15 min in ultrapure water (50 µl) to form MB‐EGFR@PS/KALA nanoparticles. AHA (5 µg), PHA (5 µg), and KALA (2 µg) were mixed in ultrapure water (50 µl) to form AHA/PHA/KALA complexes, and then added dropwise to MB‐EGFR@PS/KALA nanoparticles in ultrapure water followed by mixing for 10 min to form MB‐EGFR@PS/KALA/AHA/PHA/KALA (coded as “MB‐EGFR@MV”) nanoparticles in 100 µl of ultrapure water.

PS (30 µg), KALA (3 µg) and MB‐EGFR‐CD31 (0.07 nmol) were mixed for 15 min in ultrapure water (50 µl) to form MB‐EGFR‐CD31@PS/KALA nanoparticles. AHA (5 µg), PHA (5 µg), and KALA (2 µg) were mixed in ultrapure water (50 µl) to form AHA/PHA/KALA complexes, and then added dropwise to MB‐EGFR‐CD31@PS/KALA nanoparticles in ultrapure water followed by mixing for 10 min to form MB‐EGFR‐CD31@PS/KALA/AHA/PHA/KALA (coded as “MB‐EGFR‐CD31@MV”) nanoparticles in 100 µl of ultrapure water.

### Genome Editing by the Plasmid Delivery System and mRNA Probing by Molecular Beacon Delivery Systems in Malignant Cell Lines

Cells (A549 and EA.hy926, respectively) were seeded in a 6‐well plate (2×10^5^ cells in 2 ml of culture medium per well). After incubation for 24 h, the cell culture medium was replaced by the fresh medium (2 ml per well) containing P@MV loaded with 4 µg of plasmid for EGFR knockout or MV for comparison. The cells were treated with P@MV and MV, respectively, for 48 h to obtain edited cells and unedited cells.

For CLSM visualization, the edited cells or unedited cells were collected and seeded in a glass‐bottomed culture dish (35 mm) (1×10^5^ cells in 1 ml of culture medium). After incubation for 24 h, the culture medium was replaced by 1 ml of fresh medium containing MB‐EGFR@MV loaded with 0.07 nmol of MB‐EGFR or MB‐EGFR‐CD31@MV loaded with 0.07 nmol of MB‐EGFR‐CD31. After co‐incubation for 4 h, the cells were washed thrice by PBS. The cell nuclei were stained by DAPI, and then the cells were observed by CLSM at 400× magnification (Leica TCS SP8).

For flow cytometry analysis, the edited cells or unedited cells were seeded in a 6‐well plate (2×10^5^ cells in 2 ml of culture medium per well). After incubation for 24 h, the culture medium was replaced by 2 ml of fresh medium containing MB‐EGFR@MV loaded with 0.14 nmol of MB‐EGFR or MB‐EGFR‐CD31@MV loaded with 0.14 nmol of MB‐EGFR‐CD31. After co‐incubation for 4 h, the cells were washed with PBS thrice, digested with trypsin, collected by centrifugation, suspended in 200 µl of PBS, and subsequently analyzed by flow cytometry (Dakewe EXFLOW‐206).

### Evaluation on the Fusogenic Activity of Edited and Unedited Cancer Cells

Genome edited A549 cells and unedited A549 cells were obtained as detailed above. After that, the edited or unedited A549 cells (1×10^5^) were mixed with HUVEC (2×10^5^), and seeded in the well of a 6‐well plate, followed by co‐incubation for 48 h.

For CLSM observation, the co‐incubated A549 and HUVEC were collected, suspended in fresh culture medium (1×10^5^ cells in 1 ml medium) and seeded in glass‐bottomed culture dish (35 mm). After incubation for 24 h, the culture medium was replaced by 1 ml of fresh medium containing MB‐EGFR@MV loaded with 0.07 nmol of MB‐EGFR or MB‐EGFR‐CD31@MV loaded with 0.07 nmol of MB‐EGFR‐CD31. After co‐incubation for 4 h, the cells were washed thrice by PBS. The cell nuclei were stained by DAPI, and then the cells were observed by CLSM (Leica TCS SP8) at 400× magnification.

For flow cytometry analysis, the co‐incubated A549 and HUVEC were collected, suspended in fresh culture medium (2×10^5^ cells in 2 ml medium) and seeded in the well of a 6‐well plate. After incubation for 24 h, the culture medium was replaced by 2 ml of fresh medium containing MB‐EGFR@MV loaded with 0.14 nmol of MB‐EGFR or MB‐EGFR‐CD31@MV loaded with 0.14 nmol of MB‐EGFR‐CD31. After co‐incubation for 4 h, the cells were washed with PBS thrice, digested with trypsin, collected by centrifugation, suspended in 200 µl of PBS, and subsequently analyzed by flow cytometry (Dakewe EXFLOW‐206).

### Genome Editing by the Plasmid Delivery System and mRNA Probing by Molecular Beacon Delivery Systems in CMCs of Cancer Patients

Peripheral blood samples of NSCLC patients were provided by the Affiliated Hospital of Anhui Medical University. The investigate was approved by the Ethics Committee of Anhui Medical University with the approval numbers of 2021H001 and 82230001. The informed consent was obtained from all participants. All the experiments were conducted following pertinent guidelines. Peripheral blood from the patient P1 was collected in EDTA anticoagulant tubes. The unprocessed peripheral blood (4 ml) was placed in a 12‐well plate (1 ml of whole blood per well). Then, P@MV was added to the peripheral blood in two wells (P@MV loaded with 4 µg of CRISPR‐Cas9 plasmid in 200 µl of ultrapure water per well). For comparison, the blank vector MV was added to the peripheral blood in another two wells. After 12 h, the blood in each well was diluted with 50 ml of PBS, followed by filtration by using a 7 µm pore sized membrane filter to remove blood cells. Subsequently, isolated CMCs on the filter membrane were placed in a 12‐well plate and incubated in culture medium (2 ml per well) for 36 h to obtain genome edited CMCs treated by P@MV and unedited CMCs treated by MV.

After that, the culture medium of edited CMCs and unedited CMCs was replaced by the fresh culture medium (2 ml pre well) containing MB‐EGFR@MV loaded with 0.14 nmol of MB‐EGFR or MB‐EGFR‐CD31@MV loaded with 0.14 nmol of MB‐EGFR‐CD31. After incubation for 4 h, the culture medium was removed. CMCs on the filter membrane were washed with PBS thrice, fixed with 4% paraformaldehyde for 15 min, co‐incubated with anti‐EGFR (1:100 dilution) (Abcam) overnight, co‐incubated with the Cy3‐labelled goat anti‐mouse IgG (H+L) (1:100 dilution) (Aspen biological) for 50 min, stained with DAPI for 15 min, and observed by CLSM (Leica TCS SP8) under 600× magnification.

### Evaluation on the Fusogenic Activity of Edited and Unedited CMCs of Cancer Patients

By using the whole blood from the patient P2, genome edited CMCs and unedited CMCs were obtained as detailed above. Then, the culture medium was replaced by the fresh culture medium containing HUVEC (1×10^4^ cells in 2 ml of medium per well). After CMCs were co‐incubated with HUVEC for 48 h, the culture medium was replaced the fresh culture medium (2 ml per well) containing MB‐EGFR@MV loaded with 0.14 nmol of MB‐EGFR or MB‐EGFR‐CD31@MV loaded with 0.14 nmol of MB‐EGFR‐CD31. After incubation for 4 h, the culture medium was removed, and the cells on the filter membrane were washed with PBS thrice, followed by treatment with 4% paraformaldehyde for 15 min.

After that, the cells probed by MB‐EGFR@MV were co‐incubated with anti‐EGFR (1:100 dilution) (Abcam) and anti‐CD31 (1:150 dilution) (Affinity) overnight, co‐incubated with Cy3‐labelled goat anti‐mouse IgG (H+L) (1:100 dilution) (Aspen) and Alexa fluor 647‐labelled goat anti‐rabbit IgG (H+L) (1:200 dilution) (Beyotime) for 50 min, stained with DAPI for 15 min, and observed by CLSM (Leica TCS SP8) under 600× magnification.

The cells probed by MB‐EGFR‐CD31@MV were co‐incubated with anti‐EGFR antibody (1:100 dilution) (Abcam) and anti‐CD31 antibody (1:150 dilution) (Affinity) overnight, co‐incubated with Cy3‐labelled goat anti‐mouse IgG (H+L) (1:100 dilution) (Aspen) and CoraLite 488‐labled goat anti‐rabbit IgG (H+L) (1:100 dilution) (Proteintech) for 50 min, stained with DAPI for 15 min, and observed by CLSM (Leica TCS SP8) under 600× magnification.

### Statistical Analysis

The measurements were performed in triplicate, and data are given as mean ± standard deviation (s.d.). Statistical analysis was performed with GraphPad Prism 6.0 software using one‐way analysis of variance (ANOVA) with Tukey's multiple comparison test or two‐sided Student's t test. P < 0.05 was considered statistically significant.

## Conflict of Interest

The authors declare no conflict of interest.

## Supporting information

Supporting Information

## Data Availability

The data that support the findings of this study are available from the corresponding author upon reasonable request.
