# Supplementary material for: A Multifunctional Delivery System for Remodulating Cell Behaviors of Circulating Malignant Cells to Prevent Cell Fusion
Source: Adv Sci (Weinh). 2023 Aug 17;10(29):2303309. doi: 10.1002/advs.202303309 (PMC10582411; doi:10.1002/advs.202303309)
Supplement: Supplementary file 1 — Supporting Information [file ADVS-10-2303309-s001.pdf]

ADVANCED SCIENCE
Open Access

Supporting Information

for *Adv. Sci.*, DOI 10.1002/advs.202303309

A Multifunctional Delivery System for Remodulating Cell Behaviors of Circulating Malignant Cells to Prevent Cell Fusion

*Di Han, Xiao-Yan He, Yun Huang, Min Gao, Tao Guo, Xiao-He Ren, Xin-Ru Liao, Xue-Si Chen, Xuan Pang and Si-Xue Cheng**

Supporting Information

A Multifunctional Delivery System for Remodulating Cell Behaviors of Circulating Malignant Cells to Prevent Cell Fusion

*Di Han, Xiao-Yan He, Yun Huang, Min Gao, Tao Guo, Xiao-He Ren, Xin-Ru Liao, Xue-Si Chen, Xuan Pang, and Si-Xue Cheng**

Supplementary Materials and Methods

Materials

1-(3-Dimethylaminopropyl)-3-ethylcarbodiimide hydrochloride (EDC) was received from Shanghai Medpep Co. Ltd. (Shanghai, China). N-hydroxysuccinimide (NHS), 1-hydroxybenzotriazole (HOBt), adipic acid dihydrazide (ADH), N-succinimidyl 3-(2-pyridyldithio) propionate (SPDP), and Cell Counting Kit-8 (CCK-8) were from Sigma Aldrich. YOYO-1 was from Invitrogen. DAPI was from Beijing Solarbio Science & Technology Co. Ltd. (Beijing, China).

Cell lines

A549 cells and EA.hy926 cells were obtained from Procell Life Science & Technology Co. Ltd. (Wuhan, China). HUVEC was from China Center for Type Culture Collection. A549 cells were cultured in Ham's F-12K supplemented with 10% FBS and 1% penicillin-streptomycin. EA.hy926 cells were cultured in DMEM supplemented with 10% FBS and 1% penicillin-streptomycin. HUVEC was cultured in the specific culture medium for HUVEC. All cells were incubated at 37 °C under a humidified 5% CO_2 atmosphere.

Synthesis of AS1411 conjugated hyaluronic acid (AHA) and NLS-GE11 peptide conjugated hyaluronic acid (PHA)

After being treated with a cation exchange resin for 3 days, HA (303 μg) in 1 ml of PBS (pH 6.0) was activated with EDC and NHS for 1 h. Then, aminated AS1411 (330 μg) was added. After the reaction at room temperature for 24 h, the product was dialyzed (MWCO 10000) in deionized water for 72 h to remove the unreacted aptamer and other impurities, and then freeze dried to obtain AHA. As measured using the Quant-iT OliGreen ssDNA Assay Kit (Invitrogen), 56% aptamer fed was incorporated to the hyaluronic acid chains.

After being treated with a cation exchange resin for 3 days, HA (50 mg in 5 ml of deionized water) was mixed with ADH (112 mg). Then EDC (15 mg in 1 ml of ultrapure water) and HOBt (10 mg in 1 ml of DMSO) were added to the mixture. The pH value of the mixture was adjusted to 4.75 by adding HCl (1 M). The reaction was carried out under stirring at room temperature for 24 h. The product was dialyzed in deionized water for 72 h by a dialysis bag (MWCO 3500), and then freeze dried to obtained ADH-HA. 20 mg of ADH-HA dissolved in 5 ml of HAc/NaAc buffer (0.1 M, pH 4.75) was mixed with SPDP (3 mg) dissolved in 200 μl of DMSO, followed by stirring at 37 ºC for 4 h. The product was dialyzed using a dialysis bag (MWCO 3500) for 72 h, and then freeze-dried to obtain SPDP-HA. 2 mg of Cys-NLS-GE11 was added in the solution (pH 8.0) containing 6 mg of SPDP-HA, and then the reaction was carried out at 37 ºC in the Ar atmosphere for 24 h. The product was dialyzed in deionized water using a dialysis bag (MWCO 10000) for 72 h, and then freeze-dried to obtain PHA. As determined by the Pierce Quantitative Fluorometric Peptide Assay (Thermo Scientific), 67% peptide fed was incorporated to the hyaluronic acid chains.

Preparation of plasmid delivery systems with different composed vectors

Protamine sulfate (PS) (30 μg), KALA (3 μg) and CRISPR-Cas9 plasmid (2 μg) were mixed for 15 min in ultrapure water (50 μl) to form plasmid@PS/KALA nanoparticles. Then, hyaluronic acid (HA) (10 μg in 50 μl of ultrapure water) was added dropwise to plasmid@PS/KALA nanoparticles in ultrapure water followed by mixing for 10 min to form plasmid@PS/KALA/HA (coded as "P@V1") nanoparticles in 100 μl of ultrapure water.

PS (30 μg), KALA (3 μg) and CRISPR-Cas9 plasmid (2 μg) were mixed for 15 min in ultrapure water (50 μl) to form plasmid@PS/KALA nanoparticles. HA (5 μg) and AHA (5 μg) were mixed in ultrapure water (50 μl), and then added dropwise to plasmid@PS/KALA nanoparticles in ultrapure water followed by mixing for 10 min to form plasmid@PS/KALA/HA/AHA (coded as "P@V2") nanoparticles in 100 μl of ultrapure water.

PS (30 μg), KALA (3 μg) and CRISPR-Cas9 plasmid (2 μg) were mixed for 15 min in ultrapure water (50 μl) to form plasmid@PS/KALA nanoparticles. HA (5 μg) and PHA (5 μg) were mixed in ultrapure water (50 μl), and then added dropwise to plasmid@PS/KALA nanoparticles in ultrapure water followed by mixing for 10 min to form plasmid@PS/KALA/HA/PHA (coded as "P@V3") nanoparticles in 100 μl of ultrapure water.

PS (30 μg), KALA (3 μg) and CRISPR-Cas9 plasmid (2 μg) were mixed for 15 min in ultrapure water (50 μl) to form plasmid@PS/KALA nanoparticles. AHA (5 μg) and PHA (5 μg) were mixed in ultrapure water (50 μl), and then added dropwise to plasmid@PS/KALA nanoparticles in ultrapure water followed by mixing for 10 min to form plasmid@ PS/KALA/AHA/PHA (coded as "P@V4") nanoparticles in 100 μl of ultrapure water.

Characterizations of plasmid and molecular beacon delivery systems

900 μl of deionized water was added into the nanoparticles in 100 μl of deionized water for dilution. Then, the size and ζ potential of nanoparticles were measured by a Zetasizer (Nano ZS, Malvern Instruments).

To evaluate the encapsulation efficiency, 900 µl of deionized water was added into the nanoparticles (in 100 µl of deionized water), and then the sample was centrifuged at 10000 rpm for 30 min. The amount of unencapsulated free plasmid or molecular beacon in the supernatant was measured using a Quant-iT PicoGreen dsDNA Assay Kit or Quant-iT OliGreen ssDNA Assay Kit (Invitrogen) according to the manufacturer's protocol. Fluorescence spectroscopy (RF-5301 PC, Shimadzu) was used to determine the fluorescence intensity. The encapsulation efficiency of the plasmid or molecular beacon was calculated as

encapsulation efficiency = $(M_T - M_F) / W_T \times 100\%$

where M_T is the total mass of the plasmid or molecular beacon and M_F is the mass of the unencapsulated free plasmid or molecular beacon.

The morphology of P@MV, MB-EGFR@MV, and MB-EGFR-CD31@MV was observed by transmission electron microscopy (TEM). The samples supported on a 200-mesh copper grid were stained with phosphotungstic acid, air-dried, and then observed by TEM (JEM-2100 Plus).

Study on hybridization between molecular beacons and mRNAs in a solution

To study the dependence of fluorescence emission on the incubation time, MB-EGFR (70 nM) was co-incubated with EGFR mRNA (100 nM) in the Tris-HCl buffer (pH 8, 10 mM) with $MgCl_2$ (1 mM) and KCl (100 mM) for different times, and MB-EGFR-CD31 (70 nM) was co-incubated with EGFR mRNA (100 nM) and/or CD31 mRNA (100 nM) in the Tris-HCl buffer (pH 8, 10 mM) with $MgCl_2$ (1 mM) and KCl (100 mM) for different times.

To study the specificity of molecular beacons, MB-EGFR (70 nM) was co-incubated with EGFR mRNA or all-base mismatched mRNA (100 nM) in buffer for 3 h, and MB-EGFR-CD31 (70 nM) was co-incubated with EGFR mRNA (100 nM) and/or CD31 mRNA (100 nM) in buffer for 3 h. To confirm molecular beacons can be well encapsulated in the multifunctional vector, MB-EGFR@MV and MB-EGFR-CD31@MV were co-incubated with diverse mRNAs under the same condition.

The fluorescence emission after co-incubation was measured by a spectrofluorophotometer (RF-5301PC) with excitation at 488 nm and emission at 516 nm for detection of the emission from MB-EGFR, and excitation at 647 nm and emission at 667 nm for detection of the emission from MB-EGFR-CD31.

Cellular uptake study

To explore the cellular uptake of plasmid loaded nanoparticles, the YOYO-1 labelled plasmid was used to prepared plasmid loaded nanoparticles.

For confocal laser scanning microscopy (CLSM), cells were seeded in a glass-bottomed culture dish (35 mm) (1×10^5 cells in 1 ml of culture medium) and cultured at 37 °C for 24 h. After that, the culture medium was replaced with the fresh medium containing nanoparticles loaded with the YOYO-1 labelled plasmid (2 µg of plasmid in 1 ml of medium per well). After co-incubation for 4 h, the cells were washed thrice by PBS. The cell nuclei were stained by DAPI for 10 min, and then the cells were observed by CLSM (Leica TCS SP8) at 400× magnification.

For flow cytometry analysis, cells were seeded in a 6-well plate (2×10^5 cells in 2 ml of culture medium per well) and cultured at 37 °C for 24 h. After that, the culture medium was replaced with the fresh medium containing nanoparticles loaded with the YOYO-1 labelled plasmid (4 μg of plasmid in 2 ml of medium per well). After co-incubation for 4 h, the cells were washed thrice by PBS, digested with trypsin, collected by centrifugation, fixed with 4% paraformaldehyde, and analyzed by flow cytometry (Dakewe EXFLOW-206).

Intracellular trafficking of P@MV

Cells were seeded in a glass-bottomed culture dish (35 mm) (1×10^5 cells in 1 ml of culture medium) and cultured at 37 °C for 24 h. After that, the culture medium was replaced with the fresh medium containing P@MV loaded with the YOYO-1 labelled plasmid (2 μg of plasmid in 1 ml of medium per well). After co-incubation for particular time periods, the cells were washed thrice by PBS. The endosomes/lysosomes in cells were stained with Lysotracker for 45 min, and the cell nuclei were stained by DAPI for 10 min. Then, the cells were observed by CLSM (Leica TCS SP8) at 400× magnification.

CCK8 assay

Cells were seeded in a 96-well plate (8×10^3 cells in 100 μl of culture medium per well). After incubation at 37 °C for 24 h, the medium was replaced by the fresh medium containing free plasmid, and plasmid loaded nanoparticles, respectively. The concentration of plasmid was fixed at 2 μg ml^{-1}. After incubation for 48 h, CCK-8 was added (10 μl per well) to the treated cells, followed by incubation at 37 °C for 2 h. The absorbance of the solution was measured at 450 nm using a microplate reader (Multiskan Ascent) to determine the OD value. The cell viability was calculated as

cell viability = $OD_{treated} / OD_{control} \times 100\%$

where $OD_{treated}$ was obtained from the cells treated by a particular agent and $OD_{control}$ was obtained from untreated cells.

Western blot assay

The expression levels of proteins in untreated cells, unedited cells treated by MV and genome edited cells treated by plasmid loaded nanoparticles were determined by Western blotting.

Unedited cells and edited cells were obtained as follows. The cells were seeded in a 6-well plate (2×10^5 cells in 2 ml of culture medium per well). After incubation for 24 h, the medium was replaced by fresh medium containing plasmid loaded nanoparticles (4 μg of plasmid in 2 ml of medium per well) or MV (blank vector for comparison), and the cells were co-incubated with the plasmid loaded nanoparticles or MV for 48 h.

For Western blot analysis, the cells were washed with PBS triple times, lysed, and suspended in sodium dodecyl sulfate (SDS) sample buffer containing 1% β-mercaptoethanol. Total protein extracts were subjected to SDS-polyacrylamide gel electrophoresis. After electrophoresis, the proteins were transferred to poly(vinylidene fluoride) membranes

(Millipore). To block non-specific binding sites, the membranes were treated with TBST (Tris-buffered saline with Tween-20) containing 5% milk for 1 h. Then the membranes were incubated with the primary antibody overnight at 4 °C. After washing, the membranes were incubated with the secondary antibody for 1 h. Then an enhanced chemiluminescence system (Aspen) was used to detect the signals.

Quantitative polymerase chain reaction (qPCR) assay

The mRNA levels in untreated cells, unedited cells treated by MV and genome edited cells treated by P@MV were analyzed by qPCR.

Unedited cells and edited cells were obtained as follows. Cells were seeded in a 6-well plate (2×10^5 cells in 2 ml of culture medium per well). After incubation for 24 h, the medium was replaced by fresh medium containing P@MV (4 μg of plasmid in 2 ml of medium per well) or MV (blank vector for comparison), and the cells were co-incubated with P@MV or MV for 48 h.

For qPCR analysis, the cells were collected and the total RNA was extracted with a High Pure RNA Isolation Kit (Invitrogen). An EntiLink™ 1st Strand cDNA Synthesis Kit (ELK Biotech) was used for the first cDNA strand synthesized, and then qPCR was performed on a QuantStudio 6 Flex Real-Time PCR system (Life Technologies) with EnTurbo™ SYBR Green PCR SuperMix (ELK Biotech). The relative RNA levels were measured by the $2^{-\Delta\Delta Ct}$ method.

T7 endonuclease I (T7E1) assay

Cells were seeded in a 6-well plate (2×10^5 cells in 2 ml of culture medium per well). After incubation for 24 h, the medium was replaced by fresh medium containing P@MV (4 μg of plasmid in 2 ml of medium per well) or MV (blank vector for comparison). The cells were treated by P@MV and MV, respectively, for 48 h to obtain edited cells and unedited cells.

After that, the genomic DNA was extracted from the cells using QuickExtract DNA extraction solution (Epicentre). Genomic regions of EGFR gene were amplified by PCR and then the homoduplex products of PCR were denatured, rehybridized under stepdown annealing conditions to generate homo- and heteroduplexes. The mixture of duplexes was digested with T7E1 (NEB) that can cleave heteroduplex DNA mismatched. The products were electrophoresed on a 2% agarose gel. For comparison, the extracted genomic DNA without rehybridization was also treated by T7E1 to exclude false positive results.

DNA sequencing assay

Cells were seeded in a 6-well plate (2×10^5 cells in 2 ml of culture medium per well). After incubation for 24 h, the medium was replaced by fresh medium containing P@MV (4 μg of plasmid in 2 ml of medium per well). The cells were treated by P@MV for 48 h to obtain edited cells. After that, the genomic DNA was extracted from the cells and amplified by PCR. The product of PCR was subjected to TA cloning. The colonies were sequenced using an 3730XL DNA analyzer (Applied Biosystems).

Cell apoptosis assay

Cells were seeded in a 6-well plate (2×10^5 cells in 2 ml of culture medium per well). After incubation for 24 h, the medium was replaced by fresh medium containing P@MV (4 μg of plasmid in 2 ml of medium per well) or MV (blank vector for comparison). The cells were treated by P@MV and MV, respectively, for 48 h to obtain edited cells and unedited cells.

After that, the cells were collected and stained by an Annexin V-FITC/PI Staining Assay Kit (4A Biotech) based on the manufacturer's protocol. The stained cells were analyzed by flow cytometry (Dakewe EXFLOW-206).

Cell cycle assay

Cells were seeded in a 6-well plate (2×10^5 cells in 2 ml of culture medium per well). After incubation for 24 h, the medium was replaced by fresh medium containing P@MV (4 μg of plasmid in 2 ml of medium per well) or MV (blank vector for comparison). The cells were treated by P@MV and MV, respectively, for 48 h to obtain edited cells and unedited cells.

After that, the cell cycle was analyzed by the Cell Cycle Detection Kit (Yeasen Biotech) according to the manufacturer’s protocol. Briefly, the cells were harvested, washed with PBS, and fixed in chilled 75% ethanol for 24 h at 4 °C. Then cells were treated with the mixture solution of RNaseA and PI in dark for 30 min at 37 °C. The stained cells were analyzed by flow cytometry (Dakewe EXFLOW-206). The DNA content at different phases of the cell cycle was analyzed with ModFit LT 5.0 software.

Wound healing assay

Cells were seeded in a 6-well plate (2×10^5 cells in 2 ml of culture medium per well). After incubation for 24 h, the medium was replaced by fresh medium containing P@MV (4 μg of plasmid in 2 ml of medium per well) or MV (blank vector for comparison). The cells were treated by P@MV and MV, respectively, for 48 h to obtain edited cells and unedited cells.

After that, the cells were collected and seeded in a 6-well plate (1×10^5 cells in 2 ml of culture medium per well). When the cells were grown to 90% confluence, a 200 µl micropipette tip sterile was used to gently scratch a straight line. Then the debris was removed by PBS washing, and fresh culture medium (2% FBS) was added in the plate. After incubation for 24 h, the cells were washed with PBS, and then observed by an inverted microscope (Olympus IX73) at 200× magnification.

Transwell invasion assay

Cells were seeded in a 6-well plate (2×10^5 cells in 2 ml of culture medium per well). After incubation for 24 h, the medium was replaced by fresh medium containing P@MV (4 μg of plasmid in 2 ml of medium per well) or MV (blank vector for comparison). The cells were treated by P@MV and MV, respectively, for 48 h to obtain edited cells and unedited cells.

After that, the cells were suspended in 200 µl of serum-free culture medium and seeded into the Matrigel pro-coated upper chamber (1×10^4 cells per well in a 24-well chamber). 600 µl of culture medium (20% FBS) was added to the lower chambers to form a chemoattractant

environment. After incubation for 24 h, the noninvaded cells remained on the upper chambers were removed by cotton swabs. The cells invaded to the lower chambers were rinsed with PBS twice, fixed with 4% paraformaldehyde for 20 min, stained with 0.1% crystal violet for 10 min, and then visualized by an inverted microscope (Olympus IX73) at 200× magnification.

Verification of cellular fusogenicity of untreated A549 cells

A549 cells suspended in the serum-free culture medium were stained with Dil (Yeasen) at 1:1000 dilution at 37°C for 1 h. HUVEC suspended in the serum-free culture medium were stained with Dio (Yeasen) at 1:1000 dilution at 37°C for 1 h. After that, Dil labelled A549 ($1×10^5$ cells) and Dio labelled HUVEC ($2×10^5$ cells) were co-cultured in the well of a 6-well plate for a specific time, and then visualized by an inverted microscope (Olympus IX73) at 600× magnification.

Detection on syncytin-1 and CD44 in edited and unedited CMCs by antibody labelling

By using the whole blood from the patient P3, genome edited CMCs and unedited CMCs were obtained and probed by MB-EGFR and MB-EGFR-CD31@MV, respectively, as detailed in the Experimental Section in the main text. After that, CMCs probed by MB-EGFR@MV were co-incubated with anti-syncytin-1 (1:100 dilution) (Avivasysbio) and anti-CD44 (1:200 dilution) (Proteintech) overnight, co-incubated with Alexa fluor 647-labelled goat anti-rabbit IgG (H+L) (1:200 dilution) (Beyotime) and Cy3-labelled goat anti-mouse IgG (H+L) (1:100 dilution) (Aspen) for 50 min, stained with DAPI for 15 min, and observed by CLSM (Leica TCS SP8) under 600× magnification.

CMCs probed by MB-EGFR-CD31@MV were co-incubated with anti-syncytin-1 (1:100 dilution) (Avivasysbio) and anti-CD44 (1:200 dilution) (Proteintech) overnight, co-incubated with CoraLite 488-labelled goat anti-rabbit IgG (H+L) (1:100 dilution) (Proteintech) and Cy3-labelled goat anti-mouse IgG (H+L) (1:100 dilution) (Aspen) for 50 min, stained with DAPI for 15 min, and observed by CLSM (Leica TCS SP8) under 600× magnification.

Evaluation of the delivery efficiency and stability of the delivery system in whole blood

Peripheral blood from the patient P4 (2 ml) was placed in a 12-well plate (1 ml of whole blood per well). Then, the free YOYO-1 labelled plasmid and P@MV loaded with the YOYO-1 labelled plasmid were added to whole blood, respectively. After co-incubation for 12 h, the blood in each well was diluted with 50 ml of PBS, followed by filtration by using a 7 μm pore sized membrane filter to remove blood cells. After that, CMCs were stained with DAPI for 15 min, and observed by CLSM (Leica TCS SP8) under 600× magnification.

Detection on CMCs by antibody labelling

Peripheral blood from the patient P5 (1 ml) was placed in a 12-well plate. Then, MB-EGFR@MV loaded with 0.14 nmol of MB-EGFR was added to whole blood. After co-incubation for 4 h, the blood was diluted with 50 ml of PBS, followed by filtration by using a 7 μm pore sized membrane filter to remove blood cells. CMCs on the filter membrane were

washed with PBS thrice, fixed with 4% paraformaldehyde for 15 min, co-incubated with anti-CK8/18/19 (1:100 dilution) (Abcam) and anti-CD45 antibody (1:50 dilution) (Abcam) at 4 °C overnight. After that, the cells were carefully washed with PBS and incubated with the Cy3-labelled goat anti-mouse IgG (H+L) (1:100 dilution) (Aspen biological) and Alexa fluor 647-labelled goat anti-rabbit IgG (H+L) (Beyotime) (1:200 dilution) for 50 min, stained with DAPI for 15 min, and observed by CLSM (Leica TCS SP8) under 600× magnification.

Evaluation on viability of CMCs after genome editing

CMCs from the patient P6 were edited by P@MV as detailed in the Experimental Section in the main text. After that, the culture medium was removed, and CMCs were stained with Calcein AM (Yeasen) with a concentration of 151 μg ml^{-1}. Then, CMCs were observed by an inverted microscope (Olympus IX73) at 600× magnification.

Table S1. Size, zeta potential and encapsulation efficiency of plasmid and molecular beacon delivery systems.

Sample	Size (nm)	PDI	Zeta potential (mV)	Encapsulation efficiency (%)
P@MV	267±11	0.17	12.7±0.5	92
MB-EGFR@MV	234±5	0.18	13.2±0.4	93
MB-EGFR-CD31@MV	256±7	0.21	13.4±0.3	93
P@V1	183±2	0.04	10.8±0.1	90
P@V2	196±1	0.15	11.1±0.5	90
P@V3	217±4	0.15	11.5±0.9	91
P@V4	227±2	0.08	11.7±0.3	92

Table S2. Clinical information of non-small cell lung carcinoma (NSCLC) patients.

Patient ID	Gender	Age	TNM staging	Tumor size	Metastasis site	Purpose of study
P1	Male	47	T2N2M1	3.1 cm×2.6 cm	Lymph nodes, and bone (Multiple lung metastases)	To study genome editing on CMCs and EGFR expression before and after gene editing
P2	Female	41	T2N3M1	4.2 cm×2.9 cm	Lymph nodes, and bone (Multiple lung metastases)	To study genome editing on CMCs and cell fusion before and after gene editing
P3	Male	55	cT2N3M1	2.0 cm×3.1 cm	Lymph nodes, and bone	To study genome editing on CMCs and expression of fusion related proteins before and after gene editing
P4	Female	66	pT1bN1aM0	1.0×1.0 cm	Peripheral bronchial lymph nodes	To evaluate the delivery efficiency and stability of the delivery system in whole blood
P5	Male	62	TxN3M1c	-	Cervical lymph nodes	To verify the accurate identification of CMCs by the delivery system
P6	Male	49	cT1cN0M1a	-	pleura	To evaluate the viability of CMCs after genome editing

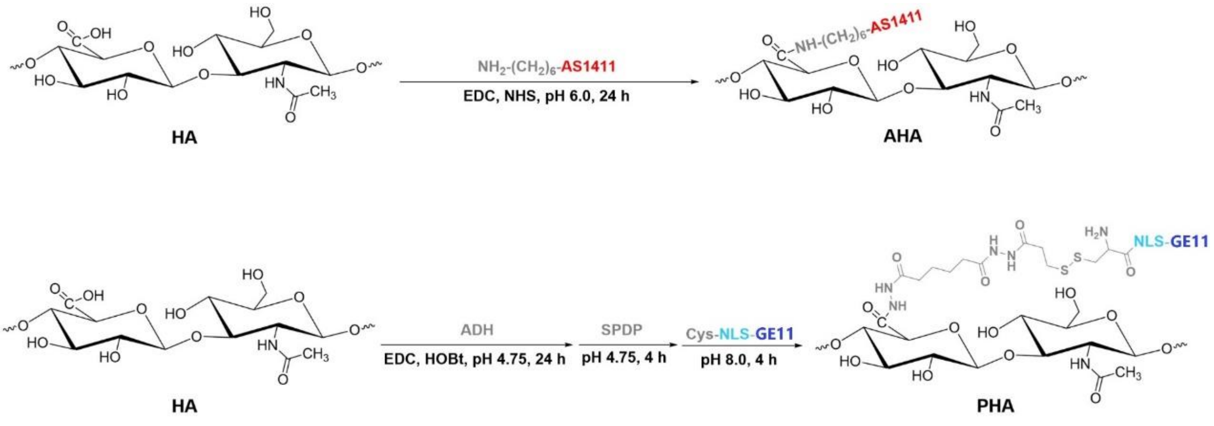

Scheme S1. Synthesis of AHA and PHA.

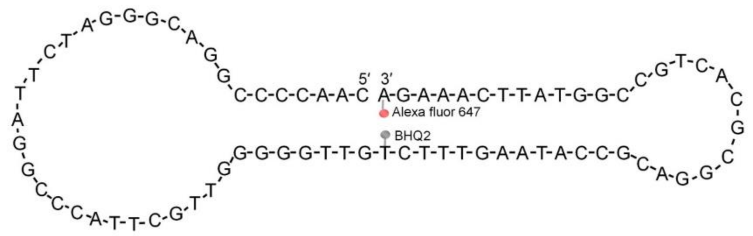

Scheme S2. Structure of the molecular beacon-based AND logic gate (MB-EGFR-CD31).

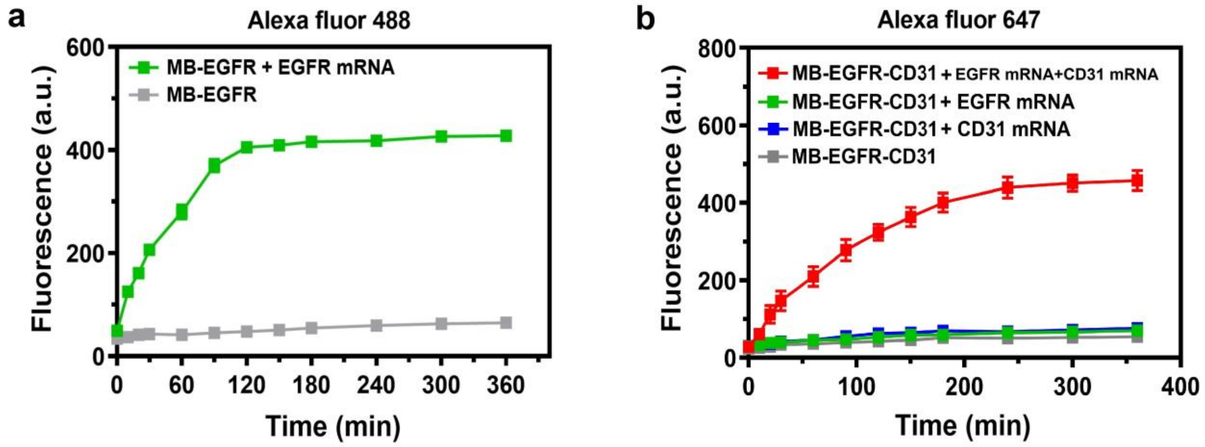

Figure S1. Study on hybridization between free molecular beacons with their target mRNAs in a buffer solution. (a) Dependence of fluorescence intensity induced by MB-EGFR on incubation time. MB-EGFR was incubated in a buffer solution in the presence or absence of EGFR mRNA (100 nM). (b) Dependence of fluorescence intensity induced by MB-EGFR-CD31 on incubation time. MB-EGFR-CD31 was incubated in a buffer solution in the presence or absence of EGFR mRNA (100 nM) and/or CD31 mRNA (100 nM). Data are given as mean ± s.d., n=3.

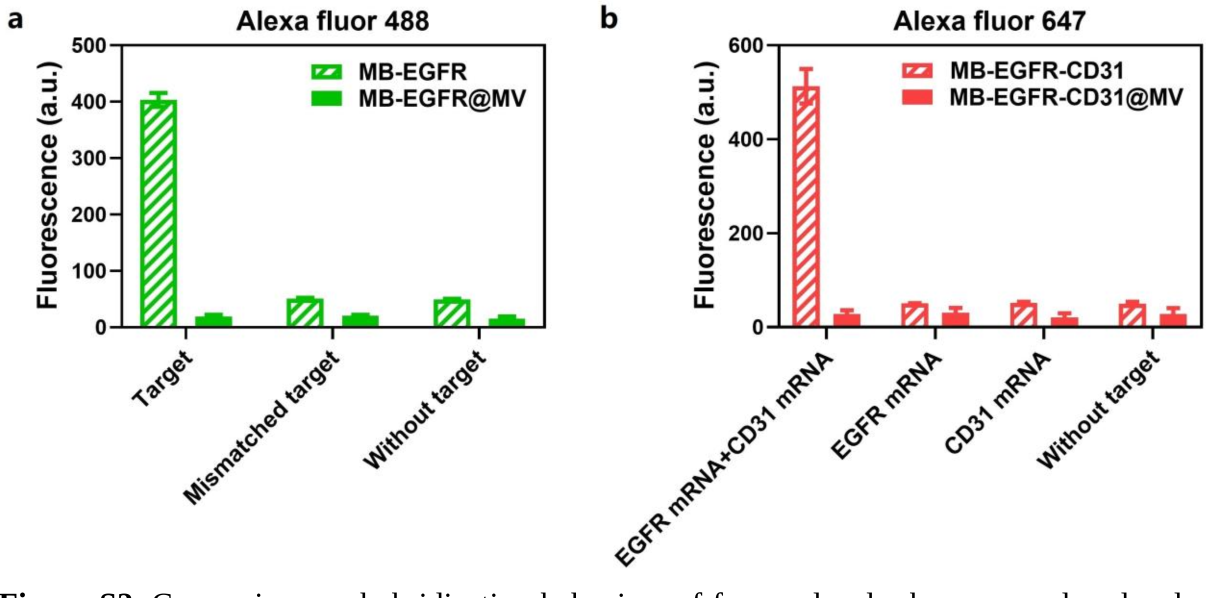

Figure S2. Comparison on hybridization behaviors of free molecular beacons and molecular beacon delivery systems in a buffer solution. (a) The fluorescence intensity induced by MB-EGFR and MB-EGFR@MV in the presence of EGFR mRNA (100 nM) and mismatched mRNA (5'-CCUGCGGUAUUCAAAGACAACCCC-3') (100 nM). (b) The fluorescence intensity induced by MB-EGFR-CD31 and MB-EGFR-CD31@MV in the presence of different mRNAs (100 nM). For comparison, the measurement was also carried out in the absence of mRNA (without target). Data are given as mean ± s.d., n=3.

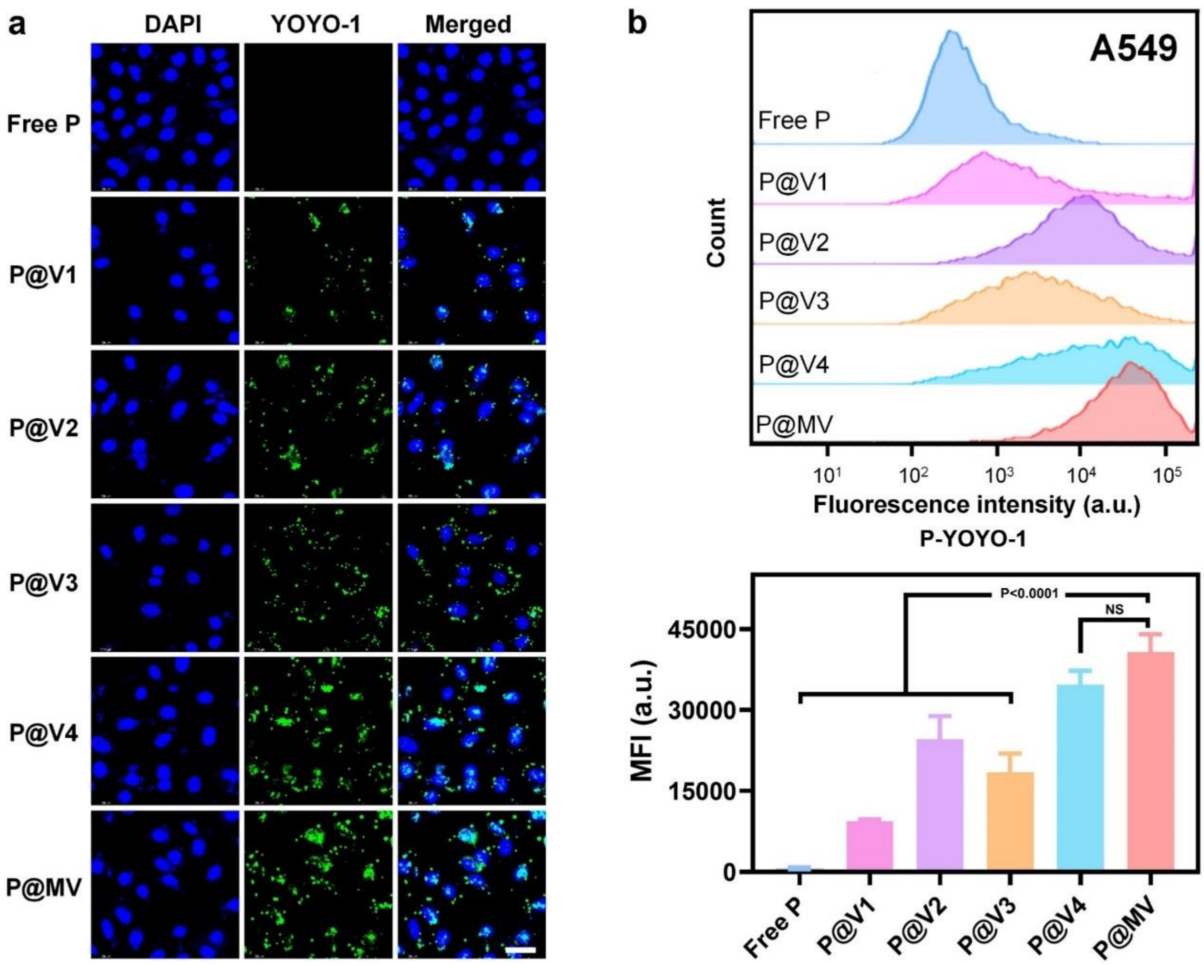

Figure S3. Study on cellular internalization of plasmid delivery systems in A549 cells. (a) CLSM images of A549 cells treated by different plasmid delivery systems (P@V1: plasmid@PS/KALA/HA, P@V2: plasmid@PS/KALA/HA/AHA, P@V3: plasmid@PS/KALA/HA/PHA, P@V4: plasmid@PS/KALA/AHA/PHA, and P@MV: plasmid@PS/KALA/AHA/PHA/KALA). CRISPR-Cas9 plasmid was labelled by YOYO-1, and cell nuclei were stained by DAPI. Scale bar: 30 μm. (b) Flow cytometry analysis on A549 cells treated by different plasmid delivery systems. A549 cells were co-incubated with plasmid delivery systems for 4 h. The cells were also treated by free plasmid (free P) for comparison. Data are given as mean ± s.d., n=3. The results were statistically analyzed using one-way ANOVA.

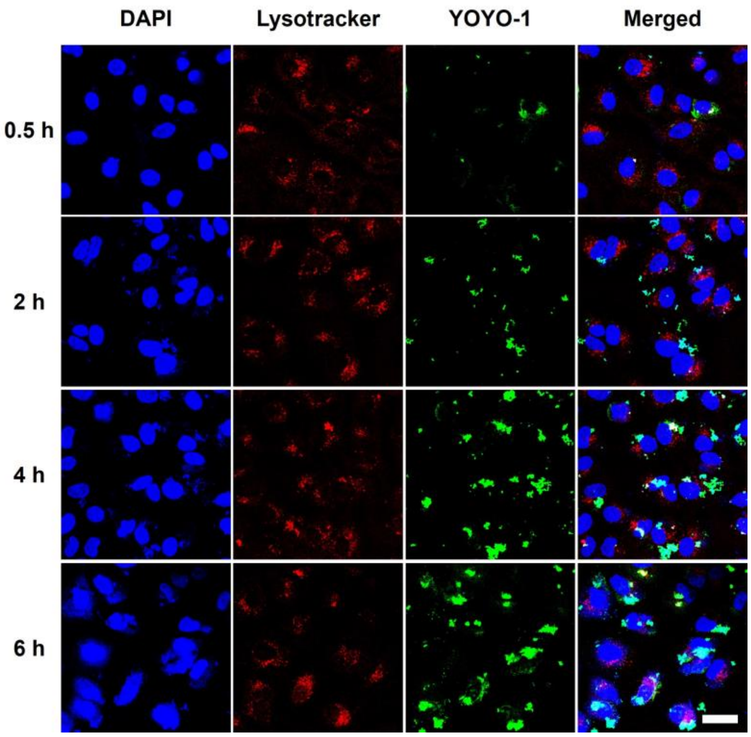

Figure S4. CLSM images of A549 cells treated by P@MV for different times. CRISPR-Cas9 plasmid was labelled by YOYO-1, endo/lysosomes were stained by Lysotracker, and cell nuclei were stained by DAPI. Scale bar: 30 μm.

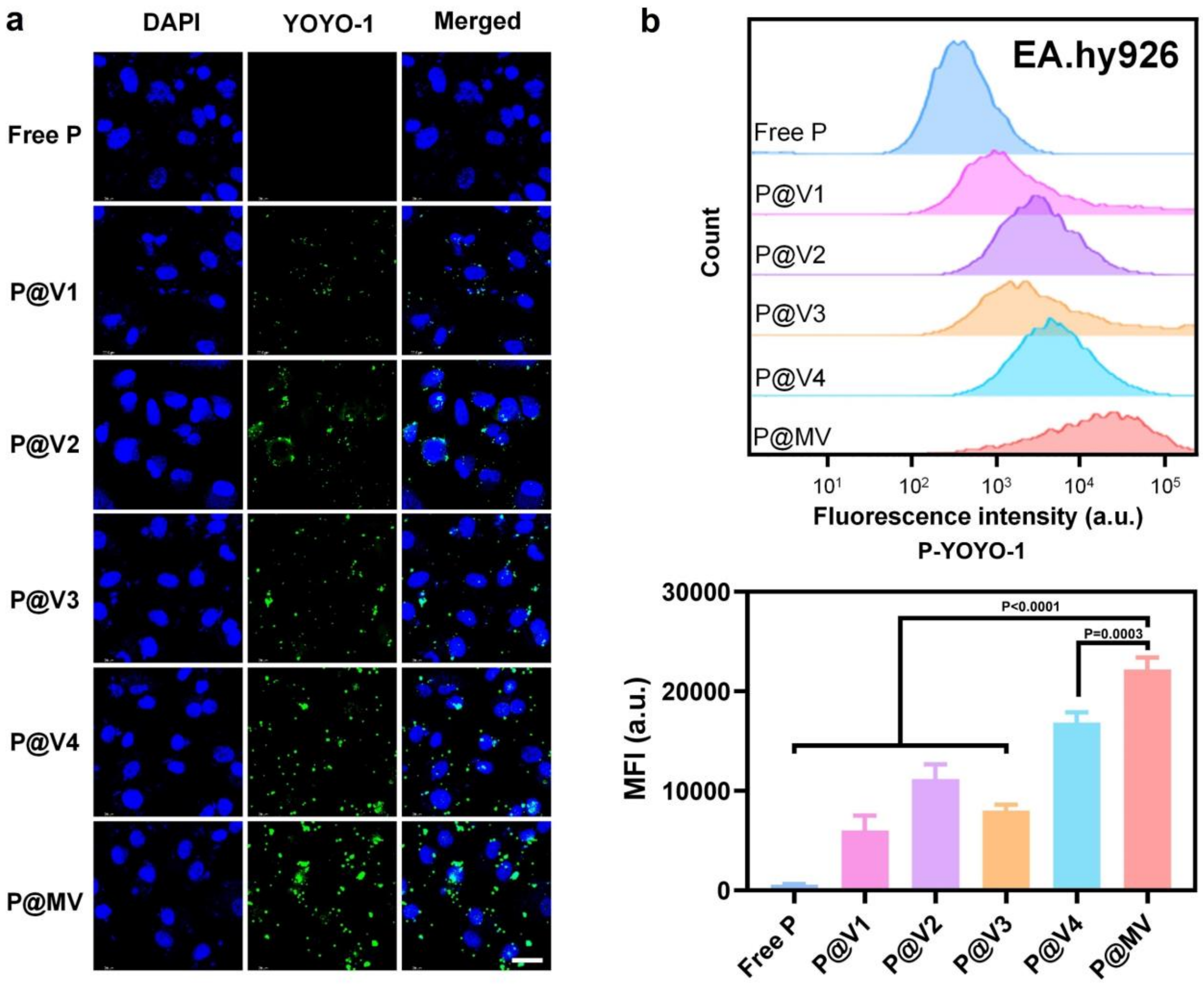

Figure S5. Study on cellular internalization of plasmid delivery systems in EA.hy926 cells. (a) CLSM images of EA.hy926 cells treated by different plasmid delivery systems (P@V1: plasmid@PS/KALA/HA, P@V2: plasmid@PS/KALA/HA/AHA, P@V3: plasmid@PS/KALA/HA/PHA, P@V4: plasmid@PS/KALA/AHA/PHA, and P@MV: plasmid@PS/KALA/AHA/PHA/KALA). CRISPR-Cas9 plasmid was labelled by YOYO-1, and cell nuclei were stained by DAPI. Scale bar: 30 µm. (b) Flow cytometry analysis on EA.hy926 cells treated by different plasmid delivery systems. EA.hy926 cells were co-incubated with plasmid delivery systems for 4 h. The cells were also treated by free plasmid (free P) for comparison. Data are given as mean ± s.d., n=3. The results were statistically analyzed using one-way ANOVA.

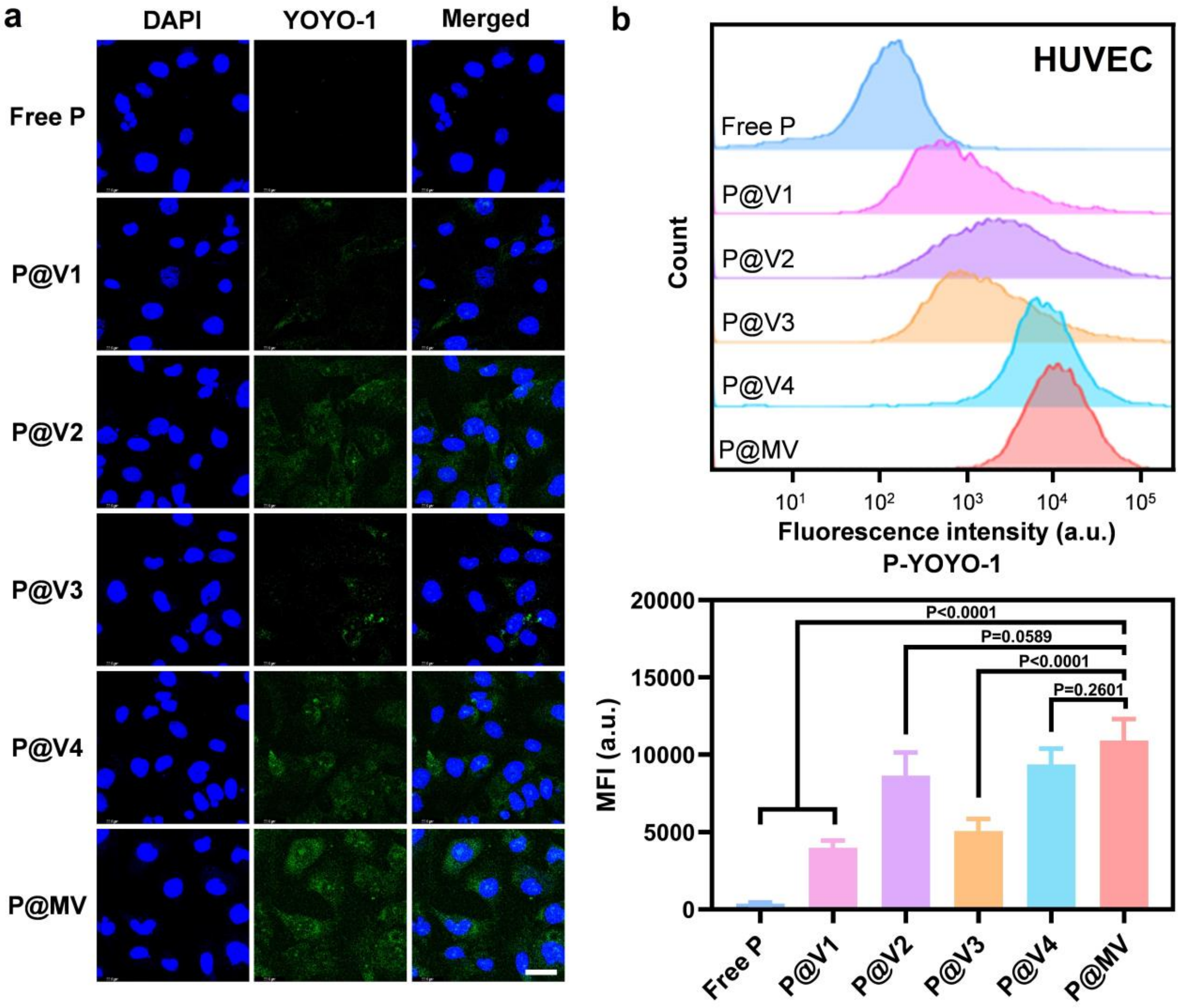

Figure S6. Study on cellular internalization of plasmid delivery systems in HUVEC. (a) CLSM images of HUVEC treated by different plasmid delivery systems (P@V1: plasmid@PS/KALA/HA, P@V2: plasmid@PS/KALA/HA/AHA, P@V3: plasmid@PS/KALA/HA/PHA, P@V4: plasmid@PS/KALA/AHA/PHA, and P@MV: plasmid@PS/KALA/AHA/PHA/KALA). CRISPR-Cas9 plasmid was labelled by YOYO-1, and cell nuclei were stained by DAPI. Scale bar: 30 µm. (b) Flow cytometry analysis on HUVEC treated by different plasmid delivery systems. The cells were co-incubated with plasmid delivery systems for 4 h. The cells were also treated by free plasmid (free P) for comparison. Data are given as mean ± s.d., n=3. The results were statistically analyzed using one-way ANOVA.

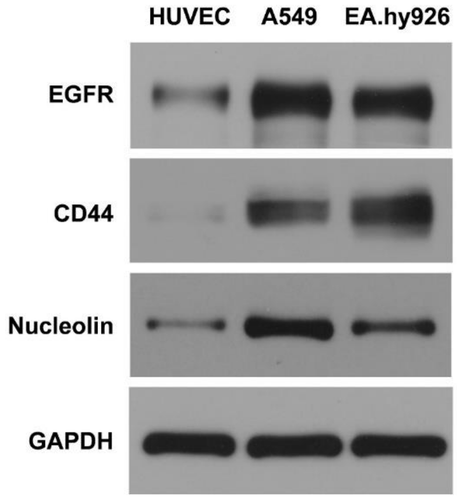

Figure S7. Western blot analysis on the expression of EGFR, CD44 and nucleolin in diverse untreated cells.

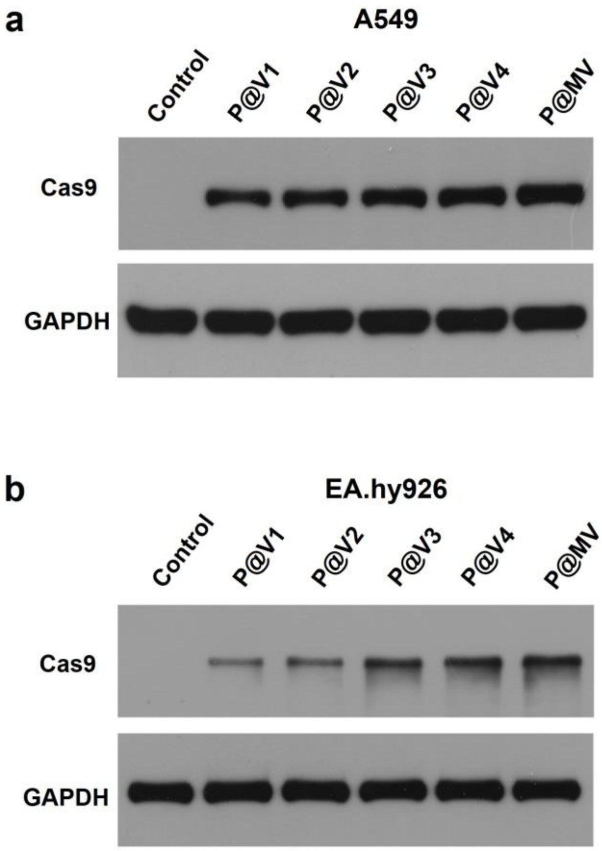

Figure S8. Western blot analysis on Cas9 expression in the cells treated by diverse plasmid delivery systems with different composed vectors. (a) A549 cells. (b) EA.hy926 cells. Untreated cells were served as a control.

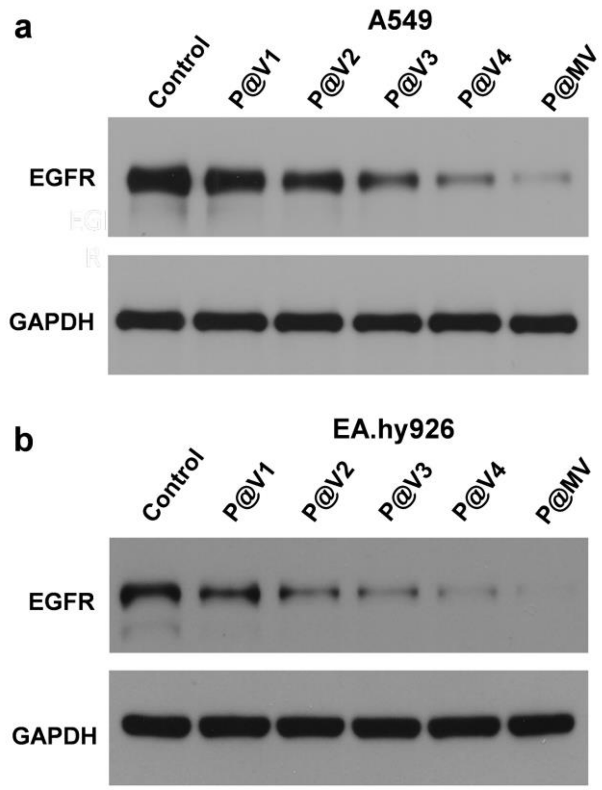

Figure S9. Western blot analysis on EGFR expression in the cells treated by diverse plasmid delivery systems with different composed vectors. (a) A549 cells. (b) EA.hy926 cells. Untreated cells were served as a control.

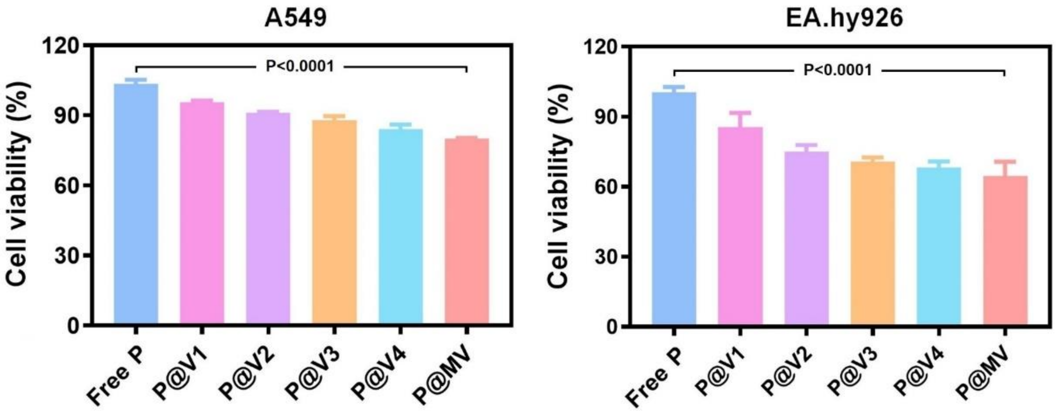

Figure S10. The cell viability after being treated by diverse plasmid delivery systems with different composed vectors. The cells were treated with plasmid delivery systems for 48 h. The cells were also treated with free plasmid (free P) for comparison. Data are given as mean ± s.d., n=3. The results were statistically analyzed using one-way ANOVA.

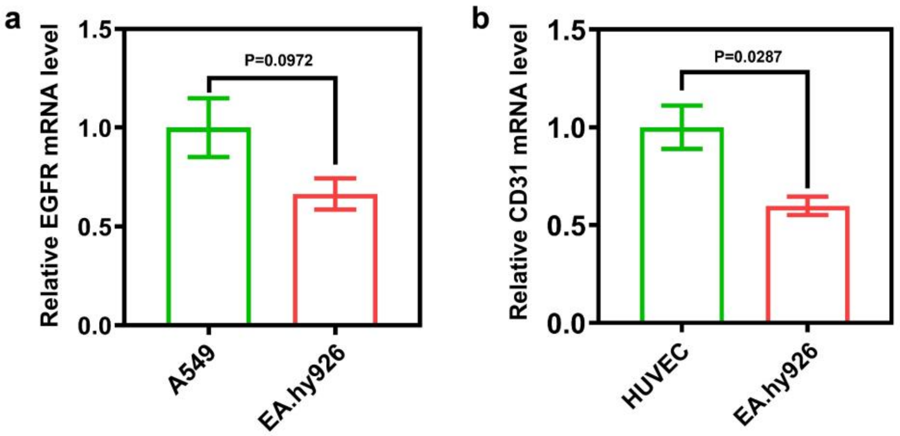

Figure S11. qPCR analysis on EGFR mRNA and CD31 mRNA in untreated cells.

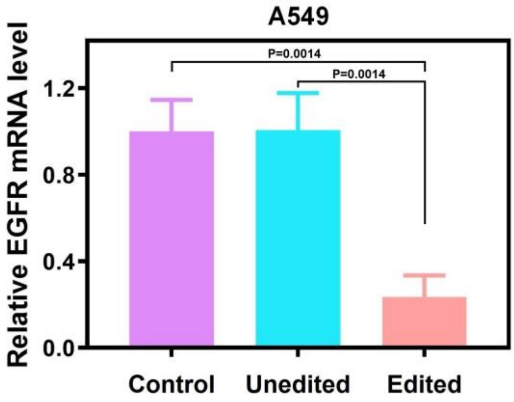

Figure S12. qPCR analysis on EGFR mRNA levels in unedited and edited A549 cells. Unedited cells were treated with the blank vector MV, and edited cells were treated by P@MV for 48 h. Untreated cells were served as a control. Data are given as mean ± s.d., n=3. The results were statistically analyzed using one-way ANOVA.

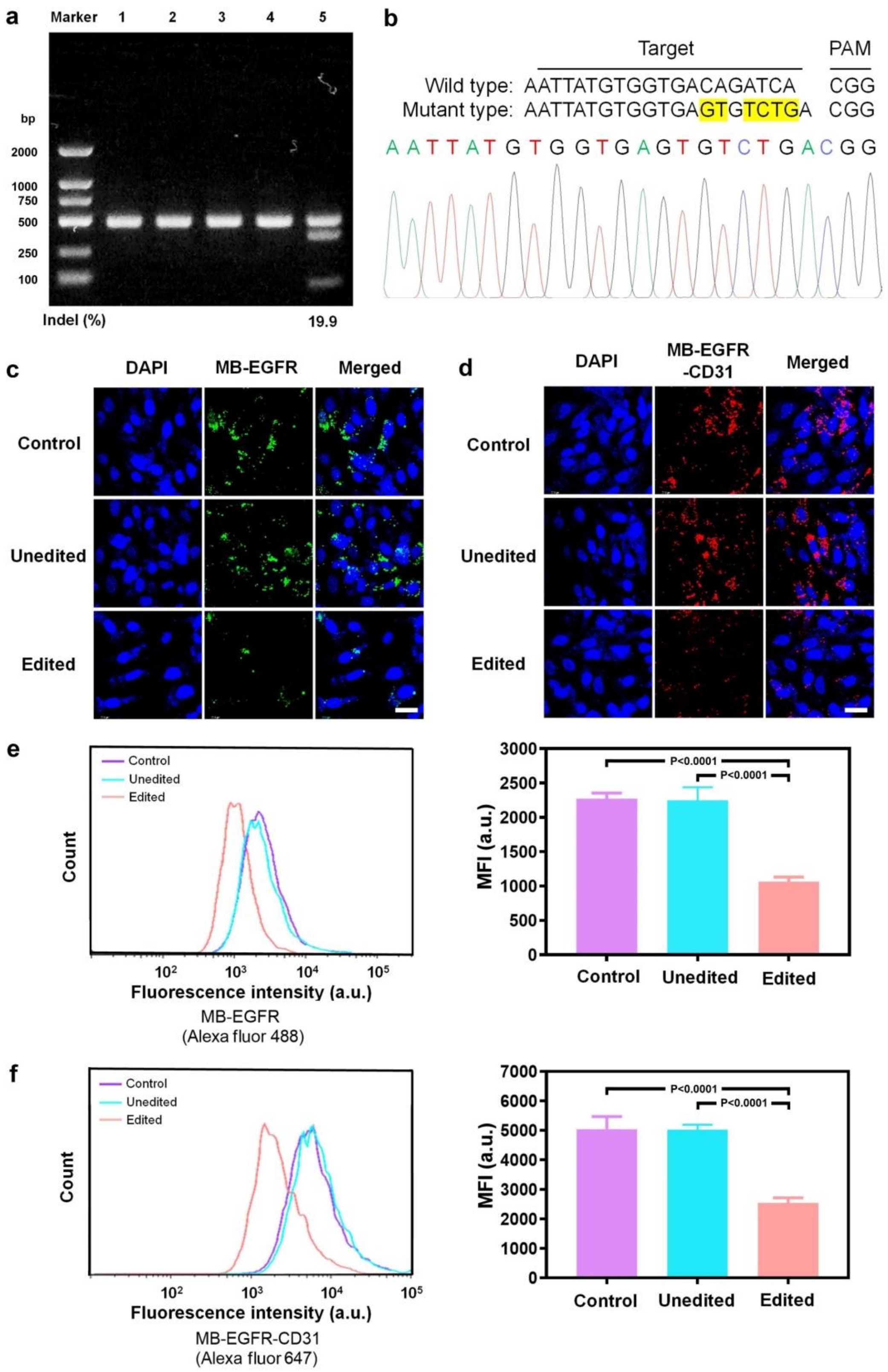

Figure S13. Genome editing on a cancer/endothelial hybrid cell line for EGFR knockout by the plasmid delivery system and probing of mRNAs by molecular beacon delivery systems. (a) T7E1 assay on the DNA mutation in EA.hy926 cells after different treatments: 1) untreated control, 2) MV treatment without rehybridization with the wild type, 3) P@MV treatment

without rehybridization with the wild type, 4) MV treatment with rehybridization with the wild type, and 5) P@MV treatment with rehybridization with the wild type. (b) The DNA sequencing result of edited EA.hy926 cells. (c) CLSM observation on EGFR mRNA probed by MB-EGFR@MV in EA.hy926 cells. Cell nuclei were stained by DAPI. Scale bar: 30 µm. (d) CLSM observation on co-existed EGFR mRNA and CD31 mRNA probed by MB-EGFR-CD31@MV in EA.hy926 cells. Cell nuclei were stained by DAPI. Scale bar: 30 µm. (e) Flow cytometry analysis on EA.hy926 cells with intracellular EGFR mRNA probed by MB-EGFR@MV. (f) Flow cytometry analysis on EA.hy926 cells with co-existed intracellular EGFR mRNA and CD31 mRNA probed by MB-EGFR-CD31@MV. Data are given as mean ± s.d., n=3. The results were statistically analyzed using one-way ANOVA. The unedited cells were treated by the blank vector MV and the edited cells were treated by P@MV for 48 h. Untreated cells were served as a control.

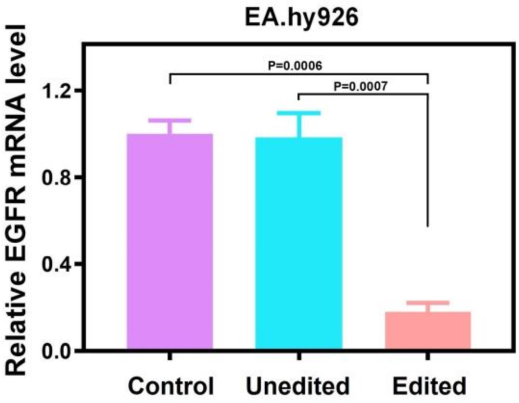

Figure S14. qPCR analysis on EGFR mRNA levels in unedited and edited EA.hy926 cells. Unedited cells were treated with the blank vector MV, and edited cells were treated by P@MV for 48 h. Untreated cells were served as a control. Data are given as mean ± s.d., n=3. The results were statistically analyzed using one-way ANOVA.

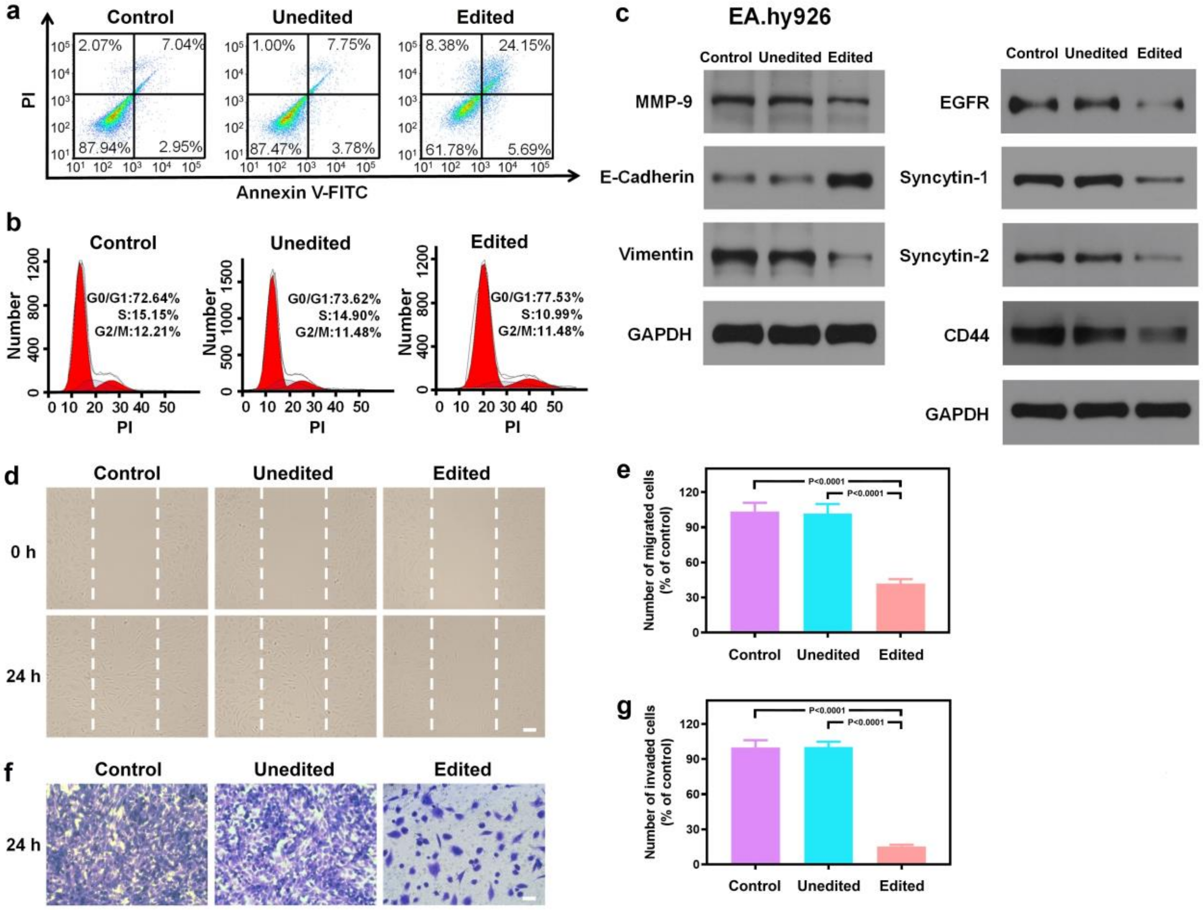

Figure S15. Inhibition of cell growth, migration, and invasion by EGFR knockout in a cancer/endothelial hybrid cell line. (a) The cell apoptosis of unedited and edited EA.hy926 cells assessed by Annexin V/PI protocol. (b) The cell cycles of unedited and edited EA.hy926 cells. (c) West blotting analysis on metastasis and fusion related proteins in unedited and edited EA.hy926 cells. (d) Wound healing assay on unedited and edited EA.hy926 cells. Scale bar: 50 μm. (e) The number of migrated unedited and edited EA.hy926 cells. Data are given as mean ± s.d., n=3. The results were statistically analyzed using one-way ANOVA. (f) Transwell invasion assay on unedited and edited EA.hy926 cells. Scale bar: 50 μm. (g) The number of invaded unedited and edited EA.hy926 cells. Data are given as mean ± s.d., n=3. The results were statistically analyzed using one-way ANOVA. Unedited cells were treated by the blank vector MV and edited cells were treated by P@MV for 48 h. Untreated cells were served as a control.

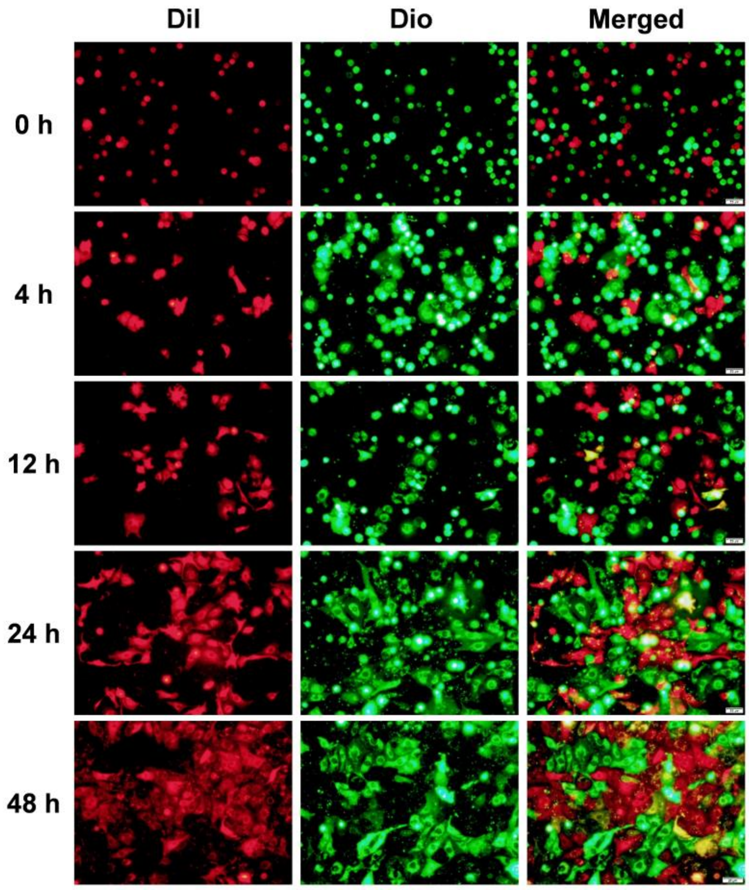

Figure S16. Study on cell fusion between cancer cells (A549) and endothelial cells (HUVEC) by staining two types of cells before co-incubation. A549 cells were stained by Dil, and HUVEC was stained by Dio. The mixed cells were observed by an inverted microscope after co-incubation for different time periods. Scale bar: 20 μm.

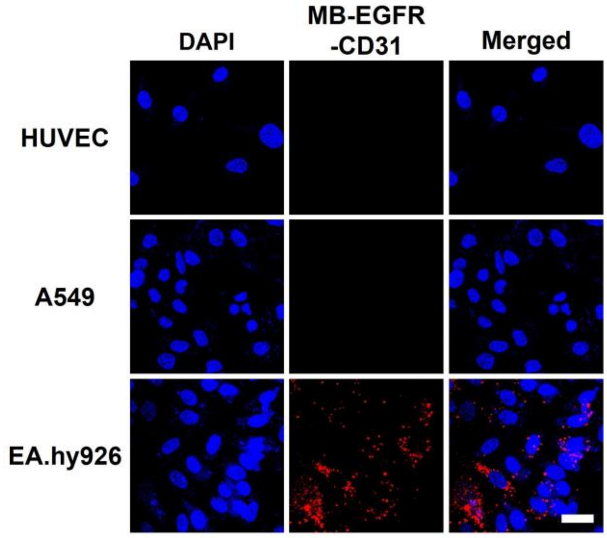

Figure S17. Logical verification of MB-EGFR-CD31@MV in diverse cells. The cells were treated with MB-EGFR-CD31@MV for 4 h, and then observed by CLSM. Scale bar: 30 μm.

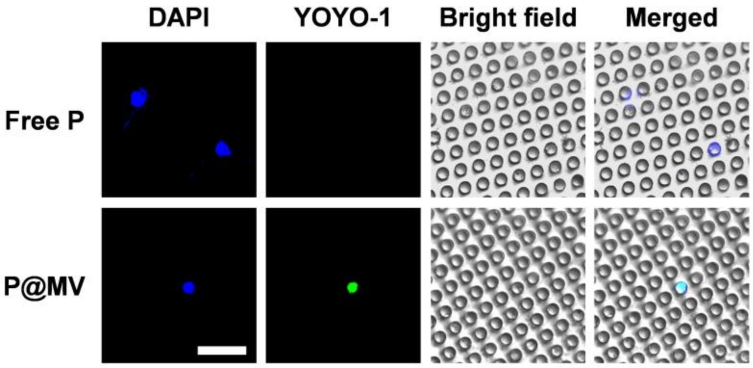

Figure S18. Verification of the delivery efficiency and stability of the delivery system in whole blood. The free YOYO-1 labelled plasmid (free P) and P@MV loaded with YOYO-1 labelled plasmid were added to the whole blood of a patient, respectively, followed by co-incubation for 12 h. After isolation by filtration, CMCs were stained with DAPI and observed by CLSM. Scale bar: 30 μm.

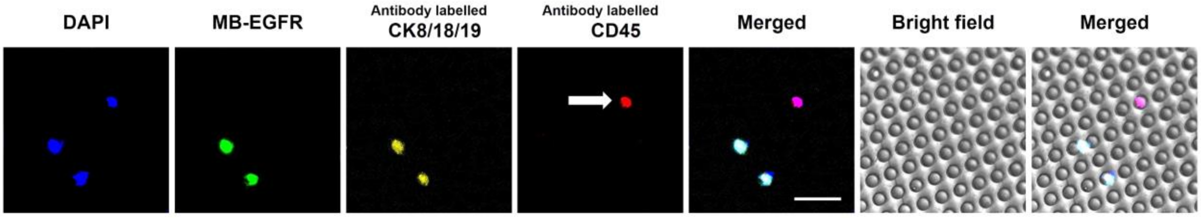

Figure S19. Verification of effective identification of CMCs by the multifunctional molecular beacon delivery system. MB-EGFR@MV was added to the whole blood from a patient, followed by co-incubation for 4 h. After isolation by filtration, the cells on the filter membrane were antibody labelled, and observed by CLSM. Scale bar: 30 μm.

As shown in Figure S19, CMCs can be easily identified by MB-EFRG@MV. No false positive signal is observed in the CD45+ blood cell indicated by the white arrow.

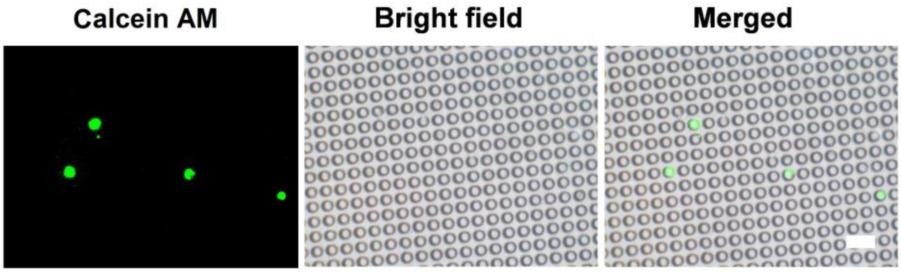

Figure S20. Study on viability of CMCs after genome editing and isolation from the whole blood. After genome editing, CMCs were stained with Calcein AM and observed by an inverted microscope. Scale bar: 20 μm.

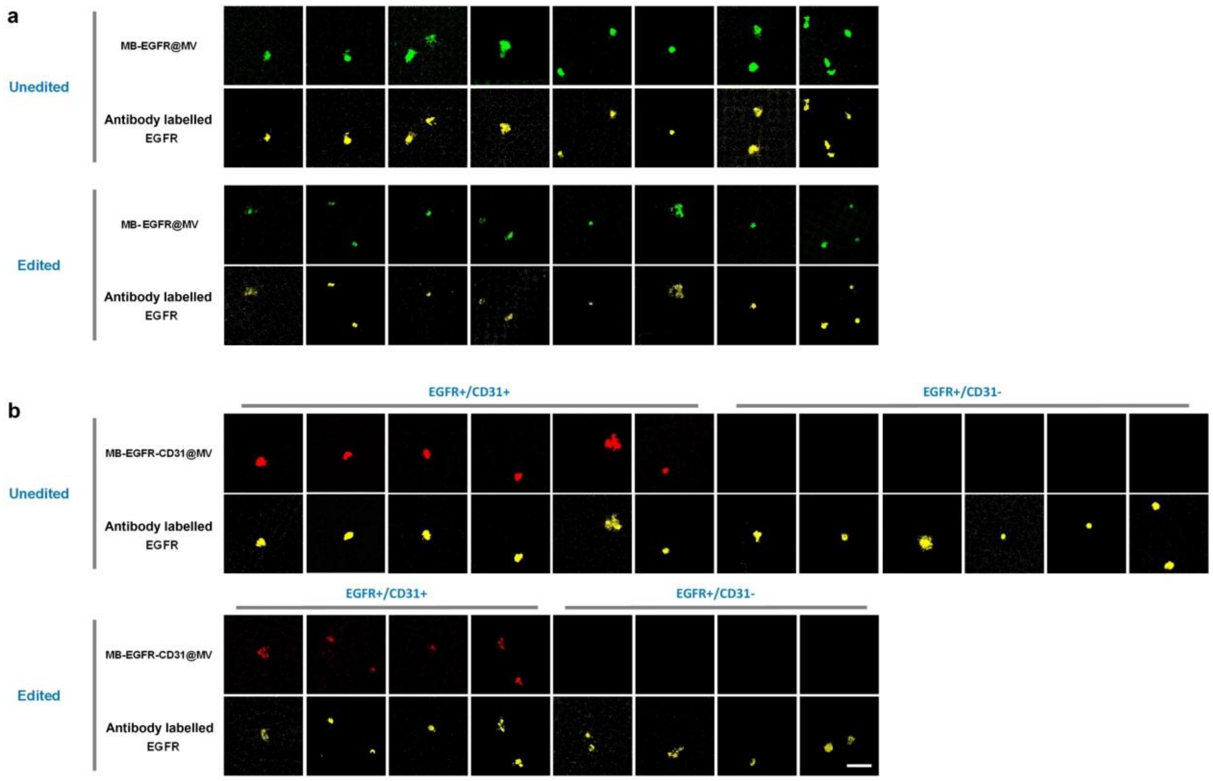

Figure S21. CLSM images of all unedited and edited CMCs from a patient (P1) as probed by molecular beacon delivery systems. (a) CLSM observation on the cells with EGFR mRNA probed by MB-EGFR@MV. (b) CLSM observation on the cells with co-existed EGFR mRNA and CD31 mRNA probed by MB-EGFR-31@MV. Cell nuclei were stained by DAPI. EGFR of CMCs was antibody labelled. Scale bar: 30 µm. The unedited CMCs were treated by the blank vector MV and the edited CMCs were treated by P@MV.

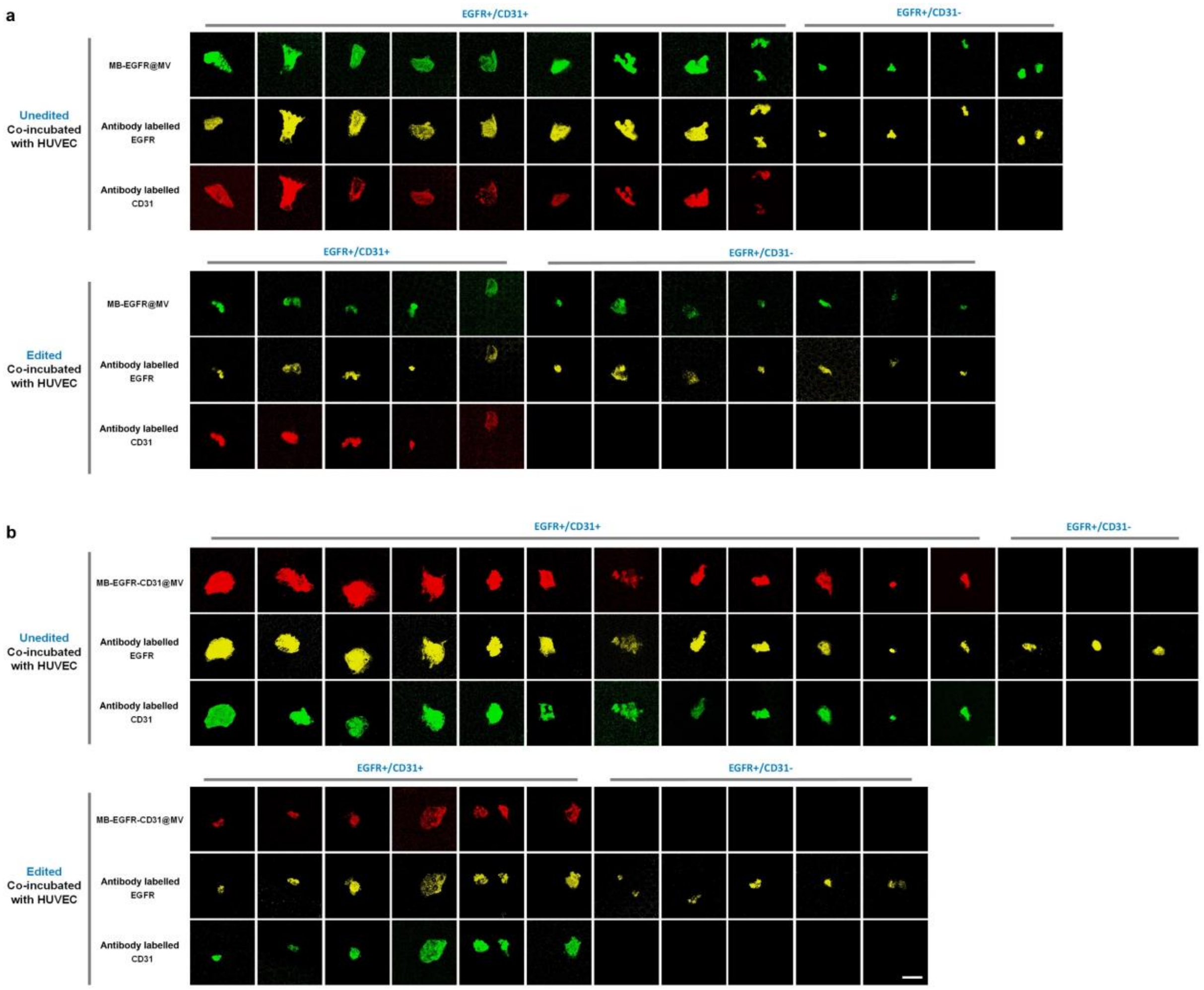

Figure S22. CLSM observation on all unedited and edited CMCs from a patient (P2) co-incubated with HUVEC. (a) CLSM images of unedited and edited CMCs co-incubated with HUVEC for 48 h and then probed by MB-EGFR@MV. (b) CLSM images of unedited and edited CMCs co-incubated with HUVEC for 48 h and then probed by MB-EGFR-CD31@MV. EGFR and CD31 of the cells were antibody labelled. Scale bar: 30 μm. The unedited CMCs were treated by the blank vector MV and the edited CMCs were treated by P@MV.
